# Maternal brain alterations based on neurotransmitter and hormone receptor distributions over six months postpartum

**DOI:** 10.1038/s41398-026-04104-4

**Published:** 2026-05-22

**Authors:** Elena Maria Losse, Negin Daneshnia, Juergen Dukart, Susanne Nehls, Natalia Chechko

**Affiliations:** 1 Department of Psychiatry, Psychotherapy and Psychosomatics, Medical Faculty, RWTH Aachen, Aachen, Germany; 2 Institute of Systems Neuroscience, Medical Faculty, Heinrich Heine University Düsseldorf, Düsseldorf, Germany; 3 Institute of Neuroscience and Medicine, Brain and Behavior (INM-7), Research Center Jülich, Jülich, Germany; 4 Institute of Neuroscience and Medicine: JARA-Institute Brain Structure Function Relationship (INM-10), Research Center Jülich, Jülich, Germany

**Keywords:** Molecular neuroscience, Psychiatric disorders

## Abstract

The long-term impact of pregnancy and post-childbirth hormonal changes on the maternal brain is yet to be fully understood. Here, we monitored 24 postpartum women longitudinally across six time points over the first 24 weeks following childbirth. By means of structural MRI, we investigated how maternal brain volume, over time and compared to nulliparous women, is associated with receptor distribution maps. While appearing to slow down toward the later time points, the voxel-wise gray matter volumes (GMV) continued to increase for at least 24 postpartum weeks, showing, from the start, a significant association with GABA_A_ and glutamate receptor distributions. The most pronounced increase was observed at three weeks postpartum, compared to childbirth, when the changes were also strongly co-localized with the cortisol, estradiol, and progesterone receptor systems. The volumetric increase from 12 to 24 weeks co-localized with areas of higher oxytocin receptor density. Compared to nulliparous controls, the maternal brains exhibited persistently smaller GMV in the amygdala, the hippocampus, the putamen and the SMA across the entire observation period. In the later postpartum phase, smaller volumes in the left hippocampus, the parahippocampal gyrus and the amygdala were found to be linked to reduced hostility toward the infant. The sustained, receptor-specific brain adaptation throughout the postpartum period was found to be complemented by the experience of maternity, likely mediated by oxytocin release. Given the robust link between these brain alterations and the GABA_A_ and glutamate receptor distributions, our findings indicate a possible role of the excitation-inhibition balance in postpartum mood regulation.

## Introduction

Pregnancy triggers profound physiological and neurobiological changes in the maternal body [1, 2], including an unprecedented increase in circulating sex steroid hormones, particularly estrogen and progesterone [1]. These hormonal shifts play a crucial role in shaping the maternal brain, modulating neurotransmission and neuroplasticity through interaction with key receptor systems, including the primary inhibitory and excitatory neurotransmitters Gamma-aminobutyric acid (GABA) and glutamate (N-methyl-D-aspartate receptor; NDMA), which are central to neuroplasticity processes [3–5]. Estrogen enhances glutamatergic neurotransmission by increasing the NMDA receptor subunit expression, the number of binding sites, and the neuronal sensitivity to synaptic input [3]. Additionally, it exerts influence on microglia cells via hormone-specific receptors, impacting neuroinflammatory responses and synaptic remodeling [6]. In contrast, progesterone and its metabolite, allopregnanolone, primarily regulate inhibitory neurotransmission via GABAergic receptors [3]. Allopregnanolone, a potent positive modulator of GABA_A_ receptors, contributes to the excitatory/inhibitory balance (E-I-balance) essential for maintaining neural stability [7]. The co-release of GABA_A_ and glutamate is central to brain function, particularly in regions such as the prefrontal cortex, the amygdala and the hippocampus [8], where the balance plays a critical role in cognitive and emotion regulation [3]. In animal models, glutamate and GABA_A_ have been found to be synchronously upregulated during the postpartum period [9], suggesting a coordinated neurochemical adaptation to motherhood. In mice, a disrupted E-I balance after simulated pregnancy has been seen to be associated with depression-like behavior, with restored E-I balance mitigating the condition [10]. Beyond these neurotransmitter systems, oxytocin emerges as another key modulator of maternal brain function. The neuropeptide enhances synaptic excitability and promotes neuroplasticity, reinforcing maternal behavior [11]. Oxytocin levels rise progressively with mother-infant interaction [12], further strengthening their attachment. Additionally, oxytocin interacts closely with the GABAergic system [13], indicating its broader role in mood regulations and stress resilience in the postpartum period. In parallel, fluctuating cortisol levels during pregnancy and the postpartum phase are also linked to the expression and function of GABA_A_ receptors [14], contributing to neural plasticity, stress resilience, and emotion regulation [15].

At the whole-brain level, previous studies have shown a linear brain volume decline during pregnancy in the cortical and subcortical structures, as well as a reduction in cortical thickness, followed by a partial postpartum recovery [16]. The brain volume reductions in pregnancy [16–19] are thought to be driven by pregnancy-related hormone fluctuations [15]. A proposed mechanism of action involves the suppression of microglia proliferation [15] through their receptors for pregnancy-associated hormones. This offers a mechanistic link between GABA_A_, glutamate, steroid hormone and oxytocin signaling systems and the neurobiological reorganization of the peripartum brain. Therefore, examining the postpartum brain volume changes in relation to the cerebral neurotransmitter receptor distributions [20, 21] may aid the identification of chemoarchitectural mechanisms underlying these structural brain changes. Using this approach, we have recently shown a link between early functional postpartum brain restorations and progesterone as well as GABA_A_ receptor distributions [22].

The corticolimbic system in particular underlies the time-sensitive and dynamic changes of the postpartum brain [23]. Compared to their nulliparous counterparts, mothers shortly after childbirth exhibited a gray matter volume (GMV) reduction in the amygdala, the hippocampus, the basal ganglia and the orbitofrontal cortex/subgenual ACC [23, 24]. Forming the corticolimbic system, the prefrontal cortices, the amygdala, and the hippocampus integrate emotion with cognition [25], and are rich in GABA_A_, glutamate and steroid receptors [3]. Interestingly, at 12 weeks postpartum, differences were still noticeable between the postpartum and nulliparous women.

Following these pregnancy-related volume reductions, a recent systematic review identified an overarching pattern of “positive” changes after childbirth as reflected in decreased brain age and regional ventricle sizes, and increased global and regional volume measures [see, 16]. The longitudinal designs typically employ highly variable time windows ranging from the very early postpartum phase to several months after childbirth, which most likely fail to systematically capture the early rapid adaptations. Our recent investigation mapped the time-sensitive developments through the first three postpartum months, identifying major changes that occur during the first six weeks following childbirth [23]. Yet, mothers are believed to reach the general pre-pregnancy physiology only around six months after childbirth [26]. It needs to be understood, therefore, how these physiological normalization processes over six months are reflected in the maternal brain adaptations.

Rather than reflecting neural loss, the structural changes are believed to reflect an adaptive reorganization of the maternal brain. Additionally, in the later postpartum months, maternal attachment may further modulate brain morphology [27, 28], becoming increasingly relevant through prolonged mother-child interaction [23, 29]. Collectively, these biological and behavioral changes during pregnancy and after childbirth are thought to underlie the dynamic adaptation of the mother’s brain, facilitating maternal behavior [30].

To examine this postpartum reorganization of the maternal brain, we performed a longitudinal neuroimaging study involving six measurements at close intervals. In addition, to understand the neurobiological reorganization behind the postpartum structural changes, we investigated their spatial association with hormone and neurotransmitter receptors. We hypothesized (I) that the maternal brain volume would increase progressively throughout the 24 postpartum weeks and (II) that this recovery would be associated with the distribution of various receptors. Specifically, we anticipated early postpartum structural changes to correlate with estrogen and progesterone receptor distributions, reflecting their acute influence on brain remodeling, and GABA_A_, cortisol and oxytocin to correlate with later postpartum changes, thus reflecting prolonged changes into motherhood. We hypothesized (III) that hormonally driven adaptations would predominate in the early postpartum phase, whereas behaviorally driven changes would become more prominent during the later postpartum period, as reflected in temporal patterns of associations with serum hormone levels, hormone- and neurotransmitter-receptor distributions, and maternal attachment to the infant. Further, (IV) we anticipated hat maternal brain volume would remain reduced relative to nulliparous women throughout the study period, with persistent differences until 24 weeks postpartum, particularly in hormone-sensitive regions such as the amygdala.

## Materials and methods

28 healthy and euthymic postpartum women were recruited in the Department of Gynecology and Obstetrics at the University Hospital Aachen. The study, approved by the Institutional Review Board of the University Hospital Aachen, conformed to the ethical standards of the Helsinki declaration from 2008 (EK-178/22). For further details of the recruitment procedure, please see the supplementary material. Structural MRI measurements were obtained within the first week of childbirth and at three, six, nine, 12 and 24 weeks postpartum. At each time point, blood samples were drawn to determine progesterone and estradiol levels. At childbirth, sociodemographic data and pregnancy- and obstetric-related information was obtained. The intention to breastfeed, and the actual breastfeeding status (yes/no) at 12 and 24 weeks postpartum were assessed. Administered at three, six, nine, 12 and 24 weeks, the Maternal Postnatal Attachment Scale (MPAS) provided a total score and three subscale scores: Absence of Hostility, Quality of Attachment and Pleasure in Interaction [31]. Of the 28 participants, three were excluded from the analyses due to a missing MRI measurement data point, and one because her mood deteriorated severely in the postpartum period. The imaging data of 24 euthymic postpartum women were included in the final analysis (mean age = 31.87, SD = 4.01, range 24 - 39 years). For details on sample size considerations and a priori power analysis for expected medium effect size, please see the supplementary material. The MPAS data were missing for three participants at 24 weeks postpartum, leaving n = 21 for the analyses with MPAS.

For cross-sectional comparisons, we included the imaging data of 24 age-matched nulliparous women measured once with no prior pregnancy and no psychiatric history from a data pool of the study center (N = 67; control group mean age = 31.54, SD = 3.85, range = 24 – 39 years). For the age-matching procedure, please refer to the supplementary material. Each participant provided written informed consent prior to enrollment in the study. From our sample, 20 postpartum women also participated in the study by Nehls et al. [23] during the first 12 postpartum weeks, and were re-assessed at 24 weeks postpartum for the present publication.

### Hormonal assays and physiological and behavioral data analysis

Maternal progesterone and estradiol blood serum concentrations were obtained prior to each MRI measurement and analyzed at the Laboratory Diagnostic Center at the University Hospital Aachen via competitive immunometric electrochemistry luminescence (for further details, see supplementary material). Progesterone and estradiol samples were missing for one participant at nine and for one at 12 weeks postpartum.

Analyses of behavioral and physiological data were conducted using SPSS 29 (IBM Corporation, Armonk, NY, USA) for Windows. The distributions of progesterone and estradiol levels were positively skewed (Shapiro-Wilk tests) and transformed logarithmically with base e to approach normality. To test for the postpartum hormonal trajectories, repeated measures analyses of variance (ANOVAs) were conducted including time points and log-transformed hormone levels as within-subject factors. In case of a significant effect of time, we additionally investigated the trajectory for the breastfeeding and non-breastfeeding mothers separately. One-way repeated measures ANOVAs were conducted for MPAS total and subscales scores. To adjust for non-sphericity (Mauchly’s test), the Greenhouse-Geisser correction was applied. The effect sizes of the significant results are reported using partial eta squared (η_p_^2^) for F-tests (small: 0.02–0.05, medium: 0.06–0.13, large: ≥ 0.14) [32]. The significance level was set at *p* < 0.05, with Bonferroni adjustments for multiple comparisons for the post-hoc pairwise comparisons.

### MRI data acquisition and analysis

The neuroimaging data of both the mothers and nulliparous subjects were acquired with a 3 Tesla Prisma MR Scanner (Siemens Medical Systems, Erlangen, Germany) at the University Hospital RWTH Aachen, using a 3-dimensional magnetization-prepared rapid acquisition gradient echo imaging (MPRAGE) sequence, to obtain t1-weighted structural images. The imaging data were preprocessed using the Computational Anatomy Toolbox (CAT12 version 2170) for SPM12 (Statistical Parametric Mapping 12) in MATLAB 2022b (MathWorks, Inc., Natick, MA). We followed the recommended default settings for cross-sectional and longitudinal analyses of CAT12 for spatial registration, segmentation and normalization. For information on cortical thickness and sulcus depth extracted for these analyses and for details on the recording protocol and preprocessing, please refer to the supplementary material.

### Voxel-based morphometry

For the cross-sectional comparison, we compared each measurement time point of the postpartum women to the nulliparous control group via independent samples t-tests, with age and total intracranial volume (TIV) as covariates [33]. To investigate longitudinal GMV changes within the postpartum group, flexible-factorial general linear models were used with the factors subject and time point, including only TIV as a covariate [33].The significant effects were further investigated via post-hoc t-contrasts. Additionally, we performed multiple linear regression analyses to examine correlations of whole-brain GMV per time point with the concurrent total and subscale scores of MPAS, as well as log-transformed progesterone and estradiol levels, while controlling for TIV and age. The statistical threshold was set at *p* < 0.05 family-wise error (FWE) voxel-level correction for multiple comparisons as our default. For the comparison between 12 and 24 weeks postpartum, as well as for the regression analyses, we additionally explored a more lenient threshold of *p* < 0.001 cluster-forming threshold and a cluster-level FWE correction in line with the methodological recommendations for detecting spatially extended effects [34]. Gray matter structures were labeled with reference to the Automated Anatomical Labeling Atlas 3 (AAL3) [35]. All results are presented in the MNI space.

### Spatial colocalization between GMV and receptor distributions

To investigate the associations of neurotransmitter receptor distributions in the brain with maternal GMV changes, we used the JuSpace toolbox version 1.5 for MATLAB [36]. We computed correlations (a) for the changes from one postpartum time point to the next in mothers, and (b) for the differences between mothers and nulliparous women, with the receptor maps of progesterone, estradiol, cortisol, oxytocin (i.e. PGR, ESR1, NR3C1/NR3C2, OXTR; from the Allen Human Brain Atlas [37]), GABA_A_ [38] and glutamate mGluR5 receptors [39]. More specifically, in case of significant contrasts in the VBM analyses, we included the respective t-statistic maps from the longitudinal or cross-sectional model to examine their relationship with the neurotransmitter maps. Per map, mean values were extracted per region of the neuromorphometrics atlas, for which we then obtained Spearman correlations between the neurotransmitter maps and the mean VBM maps. Exact p-values were computed based on spatial permutations (10 000 permutations) and adjusted for spatial autocorrelation using a gray-matter probability map included in SPM12 (TPM.nii). False discovery-rate (FDR) corrections were applied to all analyses for the number of tests performed per group/time point comparison. We further examined the intercorrelations among the neurotransmitter maps to identify cases for which caution is warranted when interpreting significant co-localization results. When significant co-localizations of GMV maps with multiple receptor maps were observed, we conducted follow-up multiple regression analyses of these receptor maps with the t-statistic map to determine whether any associations remained significant in a combined model, thereby allowing for attribution of specificity (the results are included in Supplementary Tables S9 and S10). As a specificity control analysis, we examined correlations between the GMV maps and receptor distributions with broad cortical expression but minimal theoretical relevance to postpartum adaptations or maternal functioning. We selected beta-2 adrenergic (ADRB2), serotonin 1 A (HTR1A), and dopamine (D1) receptors for this analysis. No significant associations were observed (see Supplementary Table S11), supporting the specificity of our a priori hypothesized receptor systems to postpartum-related neuroplasticity.

The neurotransmitter maps for steroid hormone, oxytocin and ADRB2 receptor distributions were constructed based on the postmortem (n = 6, 1 female, mean age = 42.5, SD = 13.38, age range = 24 - 57 years) Allen Human Brain Atlas (AHBA) mRNA gene expressions (NR3C1/NR3C2, ESR1, PGR, OXTR, ADRB2) and obtained in MNI space via (https://www.meduniwien.ac.at/neuroimaging/mRNA.html). As the sex imbalance in the AHBA sample warrants a cautious interpretation of steroid hormone receptor results in females, these analyses are best viewed as hypothesis-generating. Receptor distribution maps likely capture relatively stable features of cortical organization, whereas circulating hormone levels vary substantially over time and between individuals. Receptor–GMV associations should therefore be interpreted as reflecting regional sensitivity to hormonal signaling rather than direct correspondence with concurrent hormone concentration. The GABA_A_, glutamate (mGluR5), HTR1A and D1 receptor maps were obtained in vivo from independent groups via positron emission tomography in healthy adult subjects (GABA_A_: n = 10, mean age= 26.60 [38], SD = 7.00; and mGluR5: n = 73, mean age = 19.90, SD = 3.04 [39], HTR1A: n = 95, mean age = 28, SD = 6.9 [40]; D1: n = 13, mean age= 33, SD = 13 [41]).

## Results

Socio-demographic, pregnancy- and obstetric-related information for the postpartum sample is provided in Table S1. MPAS total scores and subscale scores remained stable throughout the postpartum period, with no significant effect of time (see supplementary material, Table S2). At childbirth, n = 22 women reported an intention to breastfeed, and n = 18 women confirmed breastfeeding both at 12 and 24 weeks postpartum. Log-transformed levels of progesterone and estradiol for the entire sample as well as for breastfeeding mothers and non-breastfeeding mothers are provided in Table S3.

**Fig. 1:**
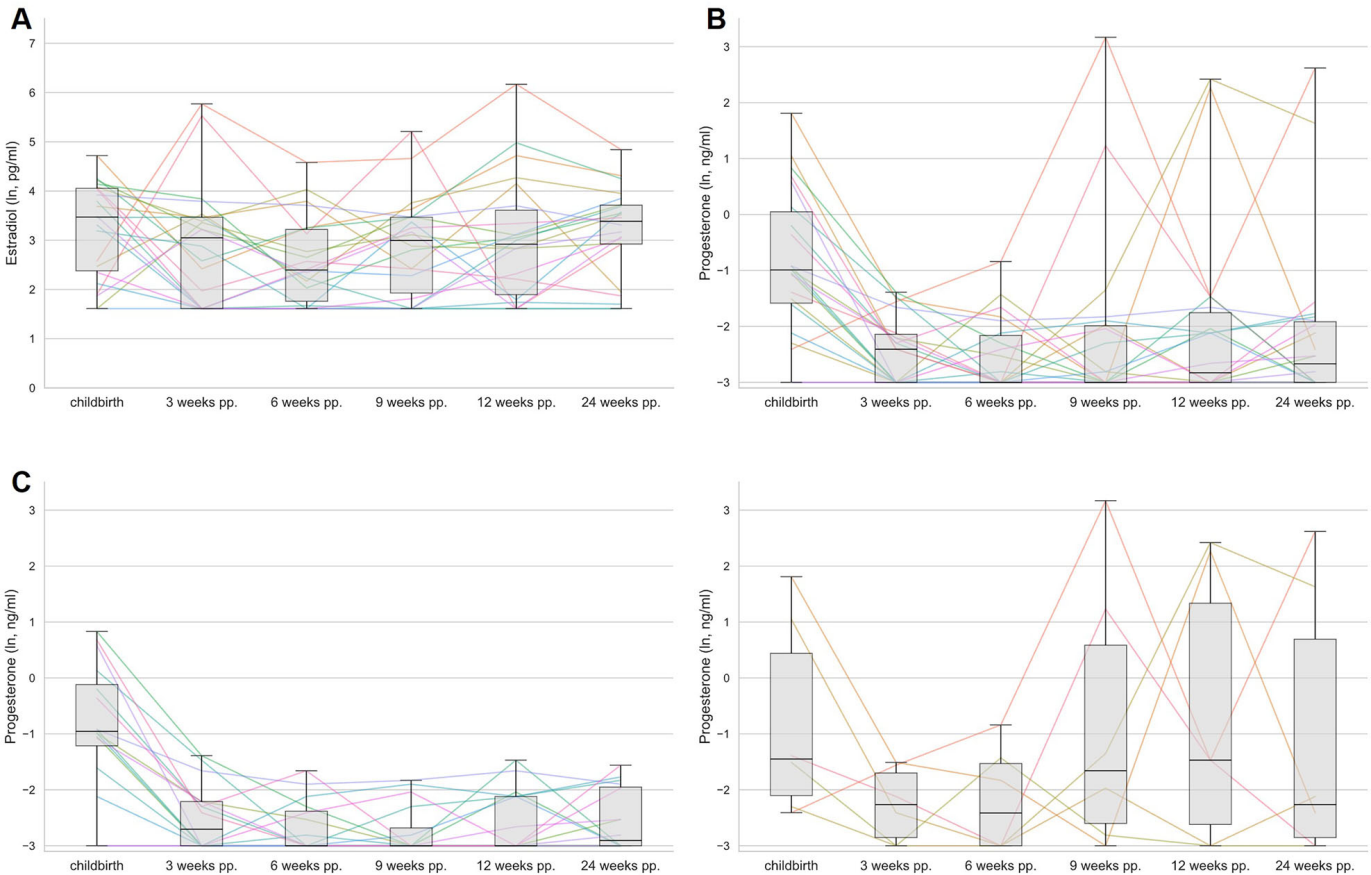
Development of natural log transformed (ln) progesterone and estradiol levels across the postpartum period. The boxplots show the group median, the 25^th^ and 75^th^ percentile and minimum and maximum per timepoint. The lines present the plasma concentration of progesterone or estradiol on the respective sampling day for the individual participants (with a complete sampling protocol, n = 22). **A** for estradiol; **B** for progesterone; **C** for progesterone in breastfeeding (n = 16; lower left) and non-breastfeeding mothers (n = 6; lower right).

Repeated-measures ANOVAs revealed no effect of time points for estradiol (*F*(3.186, 66.916) = 1.68, *p* = 0.177, η_p_^2^ = 0.074; see Fig. 1A). For progesterone, a significant effect of time was detected (*F*(2.642, 52.838) = 4.901, *p* = 0.006, η_p_^2^ = 0.197; see Fig. 1B), but no interaction of time and breastfeeding group (*F*(2.642, 52.838) = 2.622, *p* = 0.067). Nevertheless, when examining pairwise comparisons in breastfeeding mothers, progesterone levels dropped significantly between childbirth and three weeks postpartum and remained suppressed until 24 weeks postpartum, while in non-breastfeeding mothers the levels became highly variable after six weeks (Fig. 1C; Table S3).

The results for the neurotransmitter receptor map intercorrelations are provided in Table S6. They revealed an almost complete overlap of NR3C1 and NR3C2 distributions, thus warranting a discussion of cortisol more generally but not of specific receptor subtypes. Strong overlaps were also detected for estradiol receptors with NR3C1, NR3C2, and progesterone receptors, and for oxytocin receptors with progesterone receptors.

### Postpartum longitudinal GMV development in mothers and spatial association with hormone/neurotransmitter receptors

**Fig. 2:**
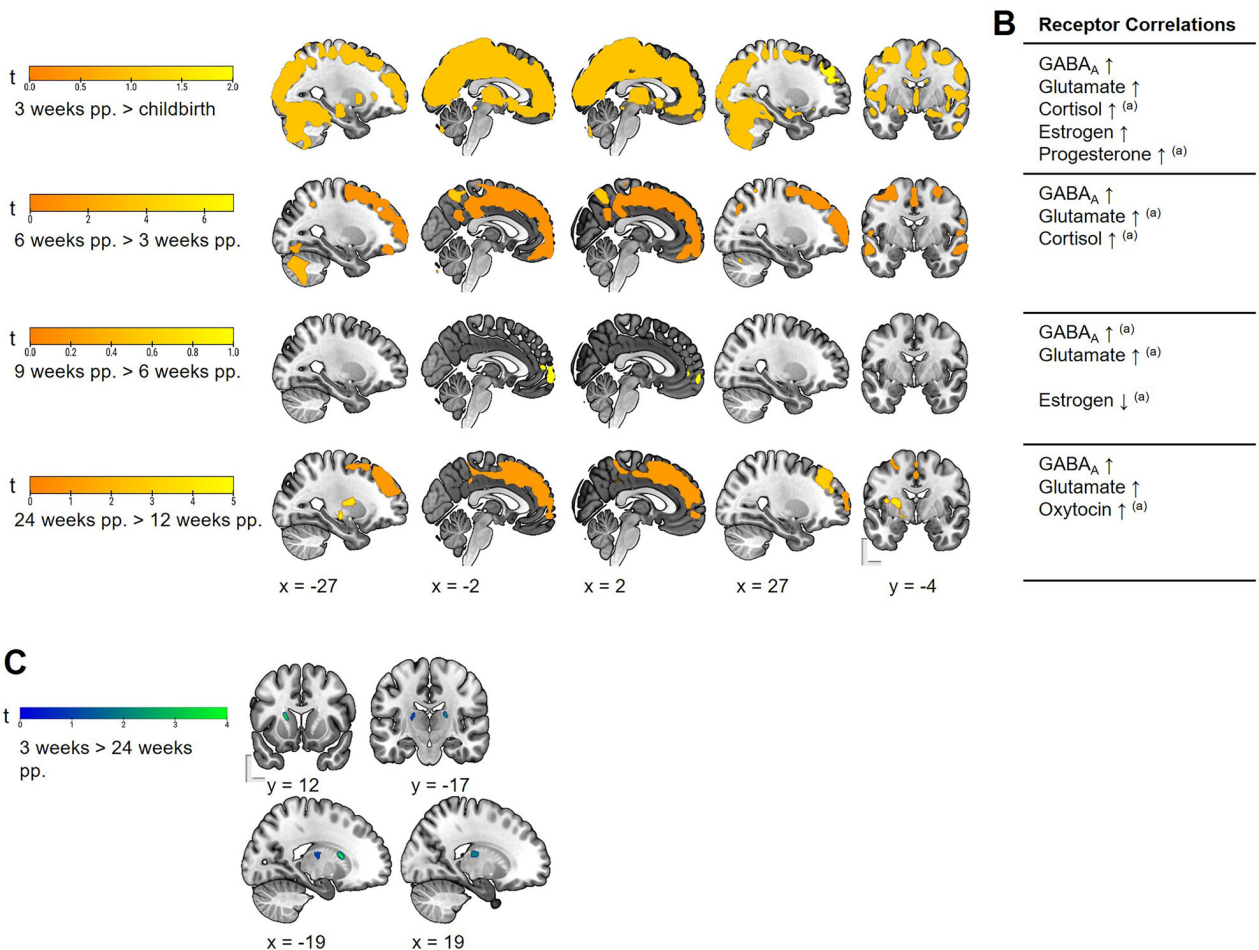
Increase of gray matter volume (GMV) and the associated receptor systems, and decrease of GMV in postpartum (pp.) women. Results are depicted with a cluster-forming threshold of *p* < 0.001 uncorrected, and *p* < 0.05 cluster-level FWE correction for better visual clarity. **A** increase in GMV in postpartum women in the first 24 weeks postpartum; **B** cerebral sex steroid and neurotransmitter receptor distributions exhibiting significant spatial correlations with GMV increase as depicted in A; ↑ = positive correlation, ↓ = negative correlation, ^(a)^ indicates associations that remained significant in the respective follow-up multiple regressions with *p* < 0.05 FDR corrected. **C** Decrease in GMV in postpartum women from three weeks to 24 weeks postpartum (*p* < 0.05 voxel-level FWE correction), encompassing the caudate nucleus, the putamen and the thalamus.

Following childbirth, maternal brains demonstrated significant and widespread GMV increases throughout the first 24 postpartum weeks (Fig. 2A, Table S4). The statistics for all longitudinal receptor analyses are provided in Supplementary Table S7. The most pronounced GMV increase was observed at three weeks postpartum compared to childbirth, when the changes were strongly co-localized with the spatial distribution of GABA_A_, glutamate, cortisol, estradiol, and progesterone receptors (Fig. 2B, Table S7). The GMV increase remained prominent and extensive until six weeks postpartum, with continued associations with GABA_A_, glutamate and cortisol receptor distributions. From six to nine weeks postpartum, the GMV increase affected fewer and smaller clusters, in fewer brain regions. Thus, the rate of GMV increase appears to slow considerably. During this period, the pattern of change shifted, maintaining spatial colocalization with GABA_A_ and glutamate receptor distributions, while becoming negatively correlated with estradiol receptor gene expression. Detailed information on regions, sizes and intensities of GMV changes from childbirth to nine weeks postpartum is provided in Supplementary Table S4.

Although between nine and 12 weeks, there was no measurable GMV change, the GMV increase continued between 12 to 24 weeks postpartum, indicating that brain adaptation persists into the later postpartum period. This increase affected the left middle and superior frontal gyri, the bilateral medial frontal gyrus, the supplementary motor area (SMA), and the pallidum, as well as the right midcingulate cortex. Using a more lenient statistical threshold (i.e. *p* < 0.001 uncorrected, with *p* < 0.05 cluster-level FWE correction), we observed GMV increases in the frontal, cingulate, parietal and temporal regions, as well as in the subcortical structures from 12 to 24 weeks postpartum (see Table S4 for detailed information). The GMV increase from 12 to 24 weeks postpartum was particularly significantly associated with the spatial distribution of GABA_A_, glutamate and oxytocin receptor gene expression, emphasizing that receptor-specific adaptations continue throughout the postpartum period.

No significant GMV decrease was detected when comparing the consecutive measurement time points. The investigation of the distal time points, which covered longer intervals, revealed a decrease in volume primarily in the basal ganglia from childbirth to 24 weeks postpartum and, additionally, in the thalamus from three to 24 weeks postpartum (see Fig. 2C and Table S4). These localized volume reductions are highly similar to our earlier findings [23], underscoring the dynamic region-specific pattern of decrease and increase in the postpartum phase.

**Fig. 3:**
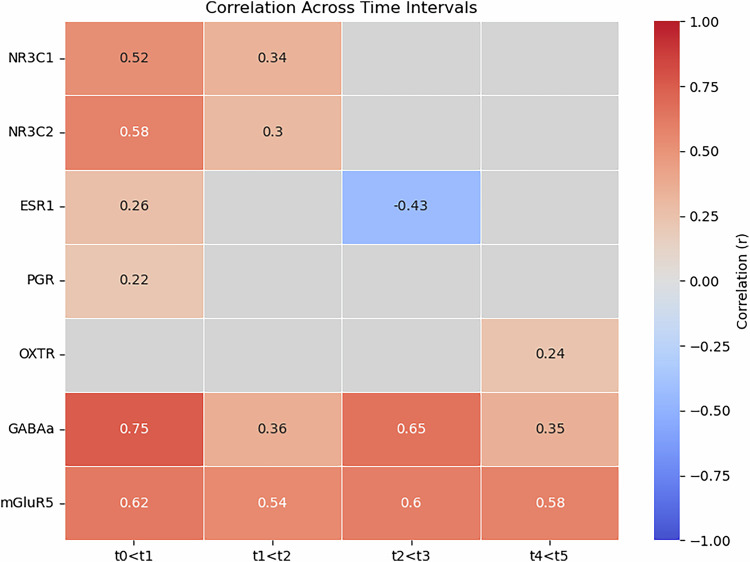
Correlation matrix between sex steroid and neurotransmitter receptor maps and maternal gray matter volume (GMV) changes in the postpartum per time point. The results depict the spearman rho per correlation with colors indicating the strength of the association, and statistically non-significant results left blank (grey) for visual clarity. NR3C1/NR3C2 = nuclear receptor subfamily 3, group C, member 1 and 2, respectively; ESR1 = Estrogen Receptor 1; PGR = progesterone receptor; OXTR = oxytocin receptor; GABA_A_ = GABA_A_ receptor; mGluR5 = metabotropic glutamate receptor 5.

Figure 3 depicts the strengths of the correlations between maternal GMV increases and the receptor systems.

### Associations of maternal brain volume with hormone concentrations and attachment

**Fig. 4:**
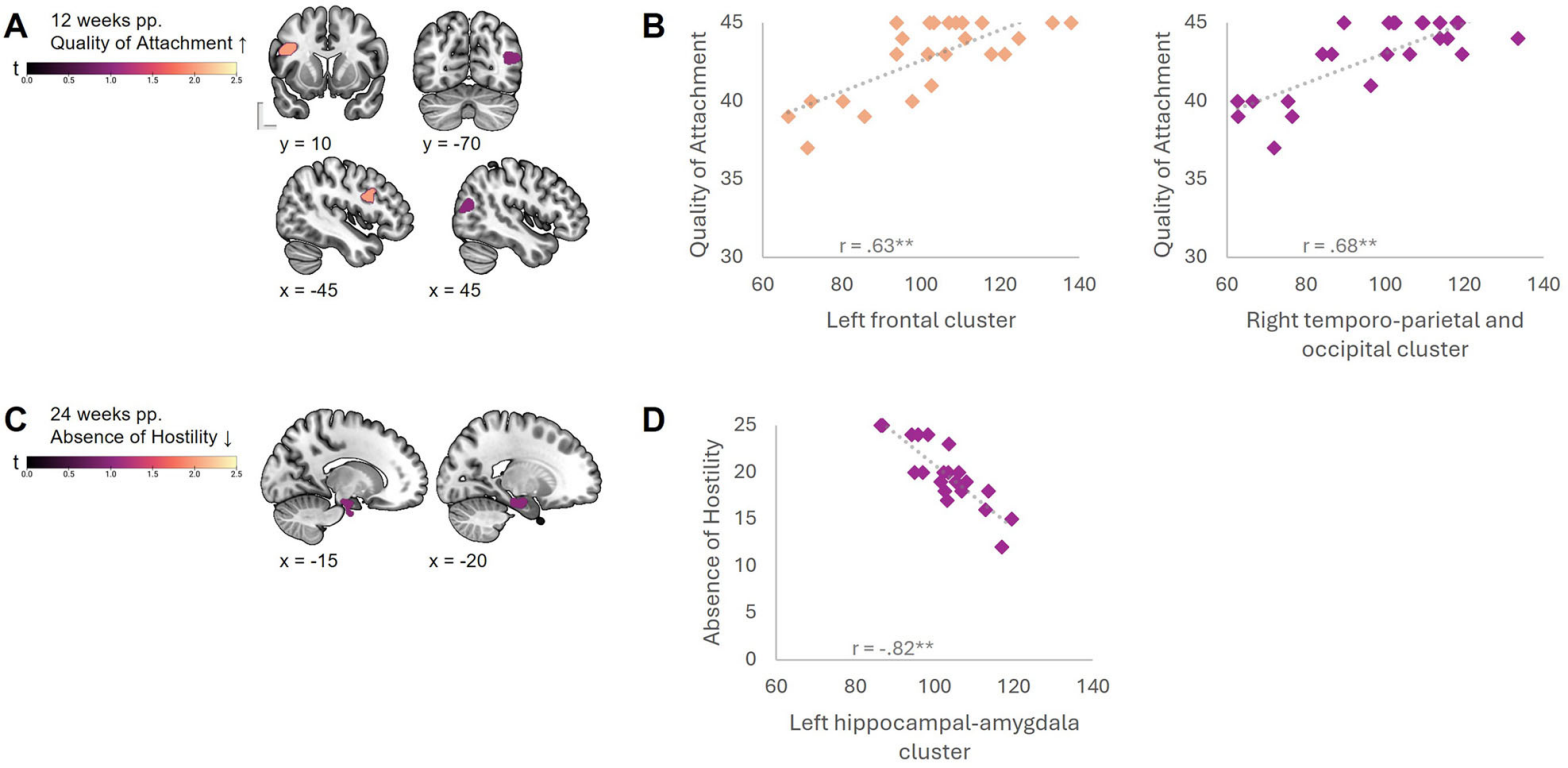
Association between maternal attachment and GMV at three and six months postpartum (pp.). Cluster-forming threshold at voxel-level *p* < 0.001. **A** Quality of Attachment at 12 weeks pp. is positively associated with maternal brain volume in two clusters. **B** Per significant cluster, volume is represented by the mean value per participant, with a trendline (dotted). Correlation with the MPAS subscale Quality of Attachment is depicted. Left panel: cluster 1 encompassing the left inferior and middle frontal gyri, and precentral gyrus. Right panel: cluster 2 encompassing the right middle temporal, middle occipital and angular gyri. **C** Absence of Hostility at 24 weeks pp. is negatively associated with maternal brain volume in one cluster. **D** For the significant cluster, volume is represented by the mean value per participant, with a trendline (dotted). Correlation with the MPAS subscale Absence of Hostility is depicted with the cluster encompassing the left hippocampus, parahippocampal gyrus and amygdala. ↑ = positive association, ↓ = negative association; ** the correlation coefficients (*r*) are significant at the *p* < 0.001 level.

Investigating the link between maternal brain changes and hormone levels more directly, and building on our prior research linking maternal brain changes to adaptive maternal behavior, we examined the association between maternal GMV and the concurrent circulating hormone levels as well as MPAS total scores and sub-scores. The multiple linear regression analyses (cluster-forming threshold at voxel-level *p* < 0.001; see Table S12) revealed significant associations parallelling the patterns from the receptor association results. Only in the early postpartum phase, at six weeks, we observed one positive correlation of GMV with log-transformed estradiol levels (left lingual gyrus, cerebellum, and vermis). A significant association with maternal attachment on the other hand emerged exclusively during the later postpartum period. Specifically, at 12 weeks postpartum, women with higher scores in quality of attachment exhibited larger GMV in the right middle temporal, angular and middle occipital gyri (see Fig. 4A and B). At 24 weeks, women with lower hostility toward their infant exhibited smaller volume in the left hippocampus, the parahippocampal gyrus and the amygdala (see Fig. 4C and D). Conversely, women reporting more pleasure in interaction had smaller left cerebellar volume (see Figure S2A and S2B), which persisted at 24 weeks postpartum (see Figure S2C and S2D).

### Maternal brain volume compared to nulliparous women

**Fig. 5:**
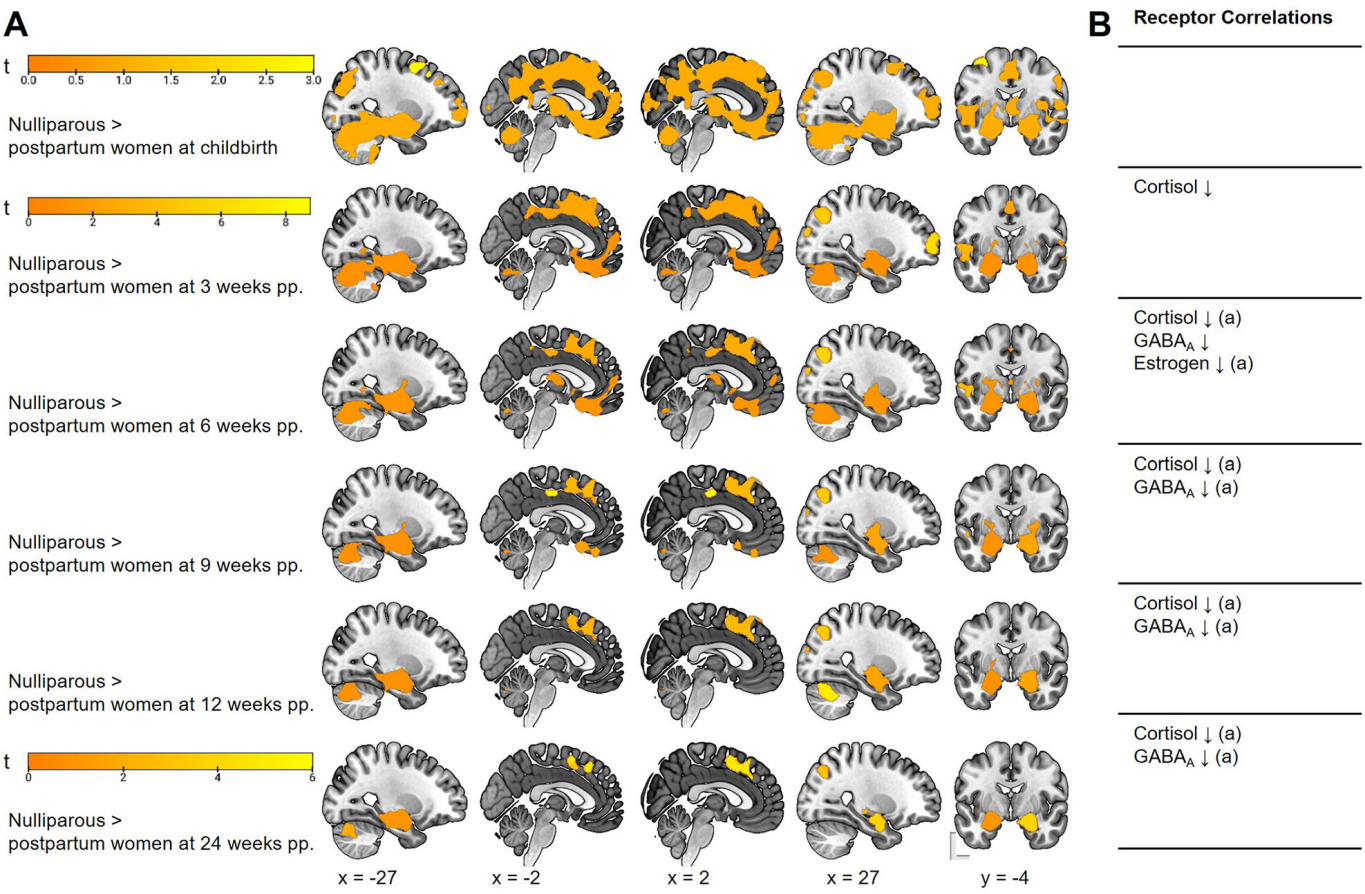
Reduced gray matter volume (GMV) in mothers across the first 24 weeks postpartum (pp.) compared to nulliparous women and the associated receptor systems. Results are depicted with a cluster-forming threshold of *p* < 0.001 uncorrected, and *p* < 0.05 cluster-level FWE correction. **A** differences in GMV between nulliparous women and mothers per postpartum time point. **B** cerebral sex steroid and neurotransmitter receptor distributions exhibiting significant spatial correlations with GMV differences as depicted in A; ↓ = negative correlation, ^(a)^ indicates associations that remained significant in the respective follow-up multiple regressions with *p* < 0.05 FDR corrected.

Finally, we explored the extent and time point of recovery of the maternal brain compared to the brains of nulliparous women. Individual t-contrasts, comparing the postpartum time points to nulliparous brains, revealed the most pronounced differences immediately after childbirth (Fig. 5A, Table S5). Although the differences persisted throughout the entire period, they progressively diminished over time particularly in the medial brain (Fig. 5A, Table S5). Nevertheless, across all time points, even until 24 weeks postpartum, smaller GMV in mothers persisted in the left amygdala, the hippocampus, the putamen, and the right SMA. To investigate receptor density distributions as a potential driver of volume recovery, Spearman correlations were performed between the volume differences per time point and the receptor density maps (Fig. 5B, Figure S1, Table S8). The analyses revealed a negative association with glucocorticoid receptor density from three weeks onwards, with GABA_A_ receptor density from six to 24 weeks, and a positive association with estradiol receptor density at six weeks postpartum. Thus, less persistent volume reductions in areas with higher density of these receptors corroborate the abovementioned longitudinal findings that postpartum recovery occurs largely in regions more sensitive to the steroid hormones.

## Discussion

In this longitudinal study, we sought to investigate maternal brain plasticity during the first six postpartum months, as well as the relationship between structural recovery and neurotransmitter receptor distributions, circulating hormone concentrations and maternal attachment.

### The most dynamic early postpartum brain recovery is linked to steroid receptor systems

The most pronounced and extended increases in maternal brain volume were found to occur in the first three weeks after childbirth, aligning temporally with a decrease of progesterone levels. These volumetric changes over the first three weeks postpartum took place primarily in regions with high densities of the GABA_A_ and glutamate receptors, as well as sex steroid and corticosteroid receptors. Regions with high progesterone and estradiol receptor densities showed greater increases only during the first three postpartum weeks, and a direct association with circulating estradiol levels was observed only at six postpartum weeks. Although the receptor-specific interpretation for female subjects is likely limited by the fact that the Allen Human Brain Atlas is based mostly on male donors, the results nonetheless indicate specific roles of the sex steroid hormones in the early postpartum brain restoration. The GMV increase was found to remain prominent and extensive until six weeks postpartum, with continued associations with GABA_A_, glutamate and cortisol receptor distributions.

### GABA_A_ and Glutamate receptor distributions and postpartum brain reorganization

While seeming to slow down toward the later time points, the maternal brain volume continued to increase at least until 24 weeks postpartum, showing persistent associations with GABA_A_ and glutamate receptor distributions. These neurotransmitter systems, influenced by estrogen and progesterone concentrations [3], regulate the brain’s excitatory/inhibitory balance, which is crucial for optimal function [42] and has long been postulated as a critical component in the pathology of depression [43]. Affecting up to 55% of women [44], postpartum mood instability is linked to the declining progesterone levels and subsequent dysregulation of GABAergic signaling. This imbalance may contribute to deficits in maternal caregiving alongside triggering anxiety- and depression-like behaviors during the postpartum period [13, 43]. Conversely, through allopregnanolone, the positive allosteric modulation of GABA_A_ receptors plays a regulatory role in postpartum mood [45], helping reduce the symptoms of postpartum disorders [46]. The regulation of mood is closely linked to the GABAergic neurocircuitry, particularly the mesocorticolimbic regions [46]. The ongoing brain volume increase at 24 weeks postpartum affects key structures within this network, including the orbitofrontal/cingulate cortex, the hippocampus and the basal ganglia. These findings highlight the importance of GABA_A_ signaling in postpartum adaptations, and its potential role in postpartum mood regulation.

### Adaptive processes associated with motherhood

To investigate the possibility of an adaptive restructuring to facilitate various aspects of motherhood, we examined the observed brain changes in relation to the indices of maternal caregiving. A link between maternal GMV and the mother’s sensitive behavior was seen to emerge at 12 and 24 weeks postpartum. A larger volume in regions associated with social cognition or visual processing and facial perception (such as the angular and middle temporal gyri) [47, 48], and a smaller volume in areas of emotion processing and stress regulation (amygdala, hippocampus) [49, 50] were found to be associated with better mother-child attachment. Furthermore, the volumetric increase from 12 to 24 weeks overlapped with areas of higher oxytocin receptor density. The oxytocin system, activated in response to reproductive stimuli, is known to reduce postpartum anxiety- and depression-like behaviors [13]. While the relationships are complex and likely reciprocal, these results suggest that mother-infant bonding likely exerts a modulatory effect on maternal brain plasticity in the later postpartum phase, possibly through prolonged interaction.

### Once a mother, always a mother: Pregnancy is associated with long-lasting volume reductions in maternal caregiving networks

Finally, we sought to investigate whether the structural changes observed shortly after childbirth, in mothers compared to nulliparous women, persisted at six months postpartum. Despite the general recovery, smaller GMV remained in the left amygdala, the hippocampus, the putamen, and the right SMA across all time points. These mesocorticolimbic structures are integral to caregiving [51], which suggests that these volumetric changes reflect an adaptive “fine-tuning” process to optimize the brain for the unique demands of motherhood [52]. The observed volume reduction beginning shortly after pregnancy and lasting throughout the entire observation period indicates that these maternal caregiving networks are hormonally primed to allow the new mother to adapt to her new role. This assumption is supported by the observation that women with lower hostility toward their infant exhibit smaller volume in the left hippocampus, the parahippocampal gyrus and the amygdala. The amygdala in particular has been seen as being integral to several networks involved in maternal bonding [53, 54]. In our earlier research, we found the amygdala volume to be the main distinguishing factor between maternal brains shortly after childbirth and those of nulliparous women [23]. Our present results suggest that the amygdala may remain permanently altered in mothers, at least during the time of intensive care for a small child. Furthermore, we observed that the volume differences between nulliparous and postpartum women co-localized with the distribution of GABA_A_ receptors, highlighting the role of GABAergic signaling in the priming of maternal brain. While one of the first studies in this field reported a return of maternal brain structure to the pre-pregnancy state by six months postpartum [55], our present results suggest the persistence of region-specific volumetric alterations, consistent with previous findings showing that such changes remain unaltered even at two to six years postpartum [28, 56].

## Limitations and conclusion

A limitation of this study is the predominantly male donor sample of the Allen Human Brain Atlas (five of six donors) [37], with sex-related differences in receptor distribution likely affecting the generalizability and accuracy of the colocalization analyses. The distributions of the two cortisol receptor subtypes overlapped too extensively to disentangle their individual effects. These limitations, however, do not undermine our findings, which demonstrate that regions with higher receptor density are critically involved in the early postpartum brain recovery. For associations between receptor maps and GMV that remained significant in the follow-up multiple regression analyses, the specificity of their effects could be demonstrated independently of the other receptor maps. In contrast, if a follow-up regression was non-significant for a receptor map, the interpretive strength of the corresponding receptor effect was limited, warranting caution for the interpretation about that specific receptor type. Receptor distribution maps likely capture relatively stable features of cortical organization, whereas circulating hormone levels vary substantially over time and between individuals. Receptor–GMV associations should therefore be interpreted as reflecting regional sensitivity to hormonal signaling rather than direct correspondence with concurrent hormone concentration. Additionally, the absence of a longitudinal nulliparous group prevents us from robustly attributing the changes to the postpartum period. However, the magnitude of the demonstrated effects across short intervals is unlikely attributable to general developmental processes. To date, our work has focused on healthy women with uncomplicated pregnancies, allowing us to characterize normative trajectories of maternal brain plasticity. However, pregnancy and the postpartum period also entail dynamic alterations in metabolic, inflammatory, endocrine, stress-related, and cardiovascular physiology, which may contribute to interindividual variability in the brain’s structural and functional adaptations. While multi-parity could alter some of the processes observed, our previous investigations could not demonstrate an effect of parity on GMV [24]. In the present sample, the differences in group size (87.5% primiparous mothers) did not support a group comparison. Lastly, the likely cellular mechanisms for the macroscopic neuronal changes remain to be confirmed [57].

Despite these limitations, the rigorous intervals of our study protocol allowed us to systematically map the time-sensitive development in postpartum women over an extended period of the postpartum phase. Unlike most previous studies, we combined the observation of this time of remarkable adult human neuroplasticity and mood fluctuations [58] with hormone and neurotransmitter receptor maps, offering insight into potential mechanistic links.

Building on our prior work examining mothers during the first three months after childbirth [see, 23], we were able to show the robustness of the observed effect with stricter statistical corrections in a larger sample. The previous absence of a significant change from nine to 12 weeks left it open whether adaptational processes might have stabilized by that time. Our extended study design demonstrated that these processes continue through at least 24 weeks postpartum, underscoring the importance of considering the later postpartum period in both research and maternal healthcare [26].

We demonstrated continuous adaptations in the maternal brain during the first six months postpartum, highlighting the role of GABAergic and glutamatergic transmission during this entire period. While the increase in brain volume in the first three postpartum weeks occurred in areas rich in sex steroid receptors, the GMV increase from 12 to 24 weeks postpartum was significantly correlated with the spatial distribution of oxytocin receptor gene expression, highlighting the receptor-specific brain adaptations throughout the postpartum period. Later, this was complemented by the experience of maternity, likely mediated by oxytocin release, exerting a modulatory influence on maternal neuroplasticity. In line with our recent findings of time-sensitive relationships between postpartum amygdala volume and hostile behavior toward the child [23], and reported protective effects of parenting in striatal and limbic regions [59–62], the present results further reinforce the notion that childbirth and motherhood drive dynamic adaptations in the maternal brain.

Taken together, our results underscore the involvement of hormones and neurotransmitters in the reorganization of the maternal brain. They also indicate an important shift from the hormonal priming of the brain during pregnancy, and its subsequent recovery during the early postpartum phase, to a continued shaping of the maternal brain through mother-child interaction throughout the later postpartum period. Future research should investigate whether disruptions in the excitation-inhibition balance contribute to postpartum depression, and whether interventions targeting the GABAergic signaling could help mitigate mood disorders in new mothers.

## Supplementary information

Supplementary Material and Methods

Figure S1

Figure S2

Figure S3

Figure S4

## Data Availability

The data of this study are not publicly available due to privacy and ethical restrictions. Data to support the findings of this study are available upon reasonable request.
