# Supplementary material for: Maternal brain alterations based on neurotransmitter and hormone receptor distributions over six months postpartum
Source: Transl Psychiatry. 2026 May 22;16:280. doi: 10.1038/s41398-026-04104-4 (PMC13197414; doi:10.1038/s41398-026-04104-4)
Supplement: Supplementary file 1 — Supplementary Material and Methods [file 41398_2026_4104_MOESM1_ESM.docx]

**Supplementary Material**

**Methods**

Recruitment procedure

We recruited 25 mothers within one to ten days of childbirth at the University Hospital Aachen. The pre-established exclusion criteria were an acute depression, or antidepressant or antipsychotic medication during pregnancy, a history of psychosis or manic episodes, as well as an abuse of alcohol, drugs or psychotropic substances. Before inclusion in the study, we screened the participants for signs of depression (prenatal) and included them only with no diagnosis of clinical depression. Upon receipt of informed consent, we obtained clinical anamnestic screenings from the participants, including demographic data, information on the pregnancy and individual and family psychiatric history.

Power analysis and sample size considerations

An a priori power analysis was conducted to estimate the sample size needed to detect longitudinal changes in brain morphometry. Based on an expected medium effect size (Cohen’s *f* = 0.25), consistent with prior longitudinal neuroplasticity research, G*Power calculations (Faul et al., 2007) indicated that a sample of 20–24 participants would be sufficient to detect within-subject effects across six repeated measurements (α = .05, power = .80). Accordingly, the final sample of 24 mothers provided adequate statistical power for the planned analyses.

Maternal Postnatal Attachment Scale (MPAS)

The MPAS is comprised of 19 items, each response weighed between one and five, compiling a maximum total score of 95, which reflects optimal maternal attachment to the infant.

Age matching of control subjects

Matching via SPSS 29 with a tolerance of 0 to achieve an exact 1:1 matching, resulted in 20 exact pairs and 4 pairs with a deviation of ± 1 - 3 years. There was no significant difference in age between the two groups (*t*(46) = 0.294, *p* = .727).

Hormone assay analysis

For the competitive immunometric electrochemistry luminescence at the Laboratory Diagnostic Center, the samples were run on a Roche Cobas e601 and on a Roche Cobas e801 with Cobas Elecsys reagents kits for estradiol and progesterone, respectively (Roche Diagnostics, Bromma, Sweden). For estradiol, the measurement interval was 5-3000 pg/ml with a coefficient of variation (CV) of 1.77 – 2.91%. For progesterone, the measurement interval was 0.05-60 ng/ml with an intra-assay CV of 2.33 -2.49%.

MRI Data Acquisition

The structural MRI measurements were obtained on average after 5.79 (SD = 2.25), 22.08 (SD = 2.28), 43.46 (SD = 2.96), 64.58 (SD = 2.8), 85 (SD = 3.46) and 174.46 (SD = 10.35) days postpartum.

The neuroimaging data were acquired with a 3 Tesla Prisma MR Scanner (Siemens Medical Systems, Erlangen, Germany), located in the University Hospital RWTH Aachen, using a 3-dimensional magnetization-prepared rapid acquisition gradient echo imaging (MPRAGE) sequence, to obtain t1-weighted structural images (4.12 min; 176 slices, TR = 2300 ms, TE = 1.99 ms, TI =900 ms, FoV= 256×256 mm2, flip angle = 9°, voxel resolution = 1×1×1mm3). We inspected all images with respect to structural abnormalities and scanner or motion artifacts, and repeated the image acquisition in case of the latter two.

The imaging data were preprocessed using the Computational Anatomy Toolbox (CAT12 version 2170) for SPM12 (Statistical Parametric Mapping 12) in MATLAB 2022b (MathWorks, Inc., Natick, MA). All images were affine registered to standard tissue probability maps by correcting individual head positions and orientations and were then transformed into Montreal Neurological Institute (MNI) space. The structural T1-weighted images acquired at each time point were spatially normalized to MNI space (resampled to a voxel size of 1.5×1.5×1.5 mm) and segmented into gray matter (GM), white matter (WM), and cerebrospinal fluid (CSF). Each image was visually inspected for potential segmentation and registration errors. Following the recommendations of the CAT12 toolbox manual (Gaser et al., 2024), a homogeneity check of the unsmoothed data revealed no outliers, allowing the GM volumes (GMVs) of all participants to be included in the subsequent analyses. Finally, the modulated GMV was smoothed with an 8-mm full-width at half-maximum (FWHM) Gaussian kernel. Measures for image quality control (CAT12 image quality rating, taking into account noise vs. signal contrast and bias field correction quality) of mothers per measurement time point are provided in supplementary table S16. To examine potential effects of delivery mode on maternal GMV, we fitted independent samples t-tests per time point, comparing women with vaginal delivery and caesarean section. As no significant GMV difference between these two groups of mothers was observed, delivery mode was not included in subsequent analyses.

Surface-Based Morphometry (SBM)

Information regarding cortical thickness was extracted via the CAT12 toolbox using the projection-based thickness (PBT) method, by selecting the surface and thickness estimation in the writing options to segment the volumes. To estimate the white matter (WM) distance, it utilizes tissue segmentation. Then, it projects the local maxima, corresponding to the cortical thickness, onto other gray matter voxels based on a neighboring relationship described by the WM distance. The PBT addresses partial volume information, sulcal blurring, and sulcal asymmetries, without requiring sulcus reconstruction (Dahnke et al., 2012). Topological defects were repaired using spherical harmonics (Yotter et al., 2011a). An algorithm was employed to achieve improved reparameterization of the cortical surface mesh, to facilitate inter-subject analysis in a common coordinate system (Yotter et al., 2011b). For the spherical registration, an adapted volume-based diffeomorphic DARTEL algorithm was utilized. Resampling and smoothing were performed with a Gaussian kernel of 12 mm FWHM for all scans. We visually inspected the data for artifacts and passed all scans through the automatic surface data homogeneity check of the CAT12 toolbox.

**Results**

Table S1. Socio-demographic information of the postpartum women.

|  | **Mean** | **SD** | **Percent** |
| --- | --- | --- | --- |
| Age | 31.87 | 1.01 |  |
| Gestational age in weeks | 39.29 | 1.76 |  |
| Child’s birth weight (g) | 3298.75 | 506.77 |  |
| Birth mode |  |  |  |
| Spontaneous |  |  | 37.5 |
| Ventouse |  |  | 16.7 |
| C-Section |  |  | 37.5 |
| Emergency C-Section |  |  | 8.3 |
| Intent to breastfeed at t0 (yes) |  |  | 91.7 |
| Breastfeeding at t4 (yes) |  |  | 75 |
| Number of children | 1.17 | 0.48 |  |
| 1 | n = 21 |  | 87.5 |
| 2 | n = 2 |  | 8.3 |
| 3 | n = 1 |  | 4.2 |
| Secondary education |  |  |  |
| Lowest (< 9 years) |  |  | 4.2 |
| Middle (10 - 12 years) |  |  | 33.3 |
| Highest (> 13 years) |  |  | 62.5 |
| Married (yes) |  |  | 83.3 |
| Single mother (yes) |  |  | 0 |

Note. t0 = within 10 days of childbirth; t4 = 12 weeks postpartum; Support at home t0 n = 21; support at home t4 n = 23.

Table S2. MPAS Descriptive statistics per time point after listwise exclusion for repeated measures ANOVAs (n= 21).

|  | **Mean** | **SD** |
| --- | --- | --- |
| MPAS 3 weeks pp. |  |  |
| Total score | 81.90 | 7.50 |
| Absence of hostility | 18.90 | 3.51 |
| Quality of attachment | 41.10 | 3.96 |
| Pleasure in interaction | 21.67 | 3.64 |
| MPAS 6 weeks pp. |  |  |
| Total score | 83.48 | 8.13 |
| Absence of hostility | 19.95 | 2.73 |
| Quality of attachment | 41.86 | 3.50 |
| Pleasure in interaction | 21.62 | 3.19 |
| MPAS 9 weeks pp. | 83.62 | 8.57 |
| Total score |  |  |
| Absence of hostility | 19.43 | 3.46 |
| Quality of attachment | 42.48 | 3.08 |
| Pleasure in interaction | 21.43 | 3.80 |
| MPAS 12 weeks pp. |  |  |
| Total score | 83.29 | 7.81 |
| Absence of hostility | 19.19 | 3.97 |
| Quality of attachment | 42.71 | 2.57 |
| Pleasure in interaction | 21.48 | 4.20 |
| MPAS 24 weeks pp. |  |  |
| Total score | 84.62 | 9.11 |
| Absence of hostility | 19.86 | 3.43 |
| Quality of attachment | 42.90 | 2.63 |
| Pleasure in interaction | 21.76 | 3.63 |

Note. MPAS = Maternal Parental Attachment Scale; pp. = postpartum.

Table S3. Natural log transformed progesterone and estradiol levels for the whole sample, and separated into breastfeeding and not breastfeeding mothers at 12 weeks postpartum.

|  | Whole sample (n = 22) | | Breastfeeding (n = 16) | | Not Breastfeeding (n = 6) | |
| --- | --- | --- | --- | --- | --- | --- |
|  | **Mean** | **SD** | **Mean** | **SD** | **Mean** | **SD** |
| Progesterone |  |  |  |  |  |  |
| Within 10 days | -0.85 | 1.32 | -0,88 | 1,17 | -0.79 | 1.79 |
| 3 weeks pp. | -2.43 | 0.60 | -2,49 | 0,59 | -2.27 | 0.66 |
| 6 weeks pp. | -2.54 | 0.64 | -2,67 | 0,46 | -2.18 | 0.95 |
| 9 weeks pp. | -2.21 | 1.54 | -2,74 | 0,44 | -0.79 | 2.47 |
| 12 weeks pp. | -2.06 | 1.54 | -2,57 | 0,55 | -0.71 | 2.46 |
| 24 weeks pp. | -2.14 | 1.47 | -2,55 | 0,55 | -1.05 | 2.50 |
| Estradiol |  |  |  |  |  |  |
| Within 10 days | 3.22 | 0.98 | 3,21 | ,97 | 3,24 | 1,09 |
| 3 weeks pp. | 2.90 | 1.22 | 2,48 | ,90 | 4,02 | 1,33 |
| 6 weeks pp. | 2.59 | 0.88 | 2,25 | ,64 | 3,49 | ,84 |
| 9 weeks pp. | 2.87 | 1.01 | 2,54 | ,80 | 3,76 | 1,05 |
| 12 weeks pp. | 3.03 | 1.24 | 2,68 | ,93 | 3,96 | 1,58 |
| 24 weeks pp. | 3.18 | 0.92 | 3,03 | ,86 | 3,58 | 1,04 |

Note. N = 22 is the sample after listwise exclusion of missing values. Pp. = postpartum.

Behavioral Data Analyses

Repeated-measures ANOVAs were performed to evaluate the effect of measurement time points on MPAS total and subscale scores. The means and standard deviations for the dependent variables are presented in Table S2.

No significant effect of time on MPAS total score (*F*(2.744, 54.87) = 1.387, *p* = .257; η_p_^2^ = .065), absence of hostility (*F*(2.739, 54.781) = .819, *p* = .479, η_p_^2^ = .039), quality of attachment (*F*(2.302, 46.031) = 1.387, *p* = .082, η_p_^2^= .113), or pleasure in interaction (*F*(4, 80) = .192, *p* = .942, η_p_^2^= .010) could be demonstrated.

**MRI results of gray matter volume analyses**

Longitudinal gray matter volume changes

The changes in brain volume of postpartum women are depicted in Figure 2A and clusters are listed in Table S4. In the first 3 weeks postpartum, the largest and most widespread GMV increases were documented in the bilateral frontal, parietal, temporal und occipital cortices and the cerebellum, including the insula, thalamus, hippocampus and parahippocampal gyrus, cingulate cortex, the left striatum and the right caudate. From week 3 to week 6 volume increases were detected primarily in the bilateral frontal cortex, the superior and middle temporal gyri, and the middle cingulate cortex, as well as in the left inferior parietal lobule and middle occipital gyrus. From 6 to 9 weeks, changing the cluster-forming threshold to *p* < .001 uncorrected, using a *p* < .05 cluster-level FWE correction revealed significant GMV increases in the bilateral medial orbitofrontal cortex, the superior medial frontal gyrus, and anterior cingulate cortex, as well as the left superior frontal gyrus. No significant volume increase was detected from 9 to 12 weeks postpartum.

Table S4. Brain regions showing gray matter volume increase in postpartum women throughout the postpartum period (t-contrast from random-effects GLM, *p* < .05, cluster-level FWE correction, unless otherwise specified, size ≥ 10 voxels).

| **Anatomical Region** | **Brodmann** | **Side** | **Size** | **T** | **x** | **y** | **z** |
| --- | --- | --- | --- | --- | --- | --- | --- |
| **3 weeks > childbirth** |  |  |  |  |  |  |  |
| Middle occipital gyrus, middle temporal gyrus, precuneus, cuneus, fusiform gyrus, calcarine sulcus, superior occipital gyrus, cerebellum 6, inferior occipital gyrus, postcentral gyrus, cerebellum crus1, inferior temporal gyrus, superior temporal gyrus, lingual gyrus, calcarine sulcus, cerebellum crus2, cerebellum 8, cerebellum 7b, inferior parietal lobule, cerebellum crus1, supramarginal gyrus, midcingulate cortex, angular gyrus, posterior cingulate cortex, superior parietal lobule, precentral gyrus, cerebellum 4 5, parahippocampal gyrus | 1, 2, 3, 4, 5, 6, 7, 13, 17, 18, 19, 20, 21, 22, 23, 29, 30, 31, 37, 39, 40 | L/R | 43985 | 8.14 | -46.5 | -72 | 0 |
| Midcingulate cortex, supplementary motor area, anterior cingulate cortex, superior medial frontal gyrus, paracentral lobule | 4, 5, 6, 8, 9, 10, 23, 24, 32, 31 | L/R | 6914 | 7.33 | -12 | -30 | 45 |
| Rolandic operculum, insula, superior temporal gyrus, supramarginal gyrus, heschl gyrus, inferior frontal gyrus p. orbitalis, inferior frontal gyrus p. triangularis, inferior frontal gyrus p. opercularis, postcentral gyrus | 6, 13, 22, 40, 41, 42, 43, 44, 45, 47 | R | 2851 | 7.10 | 58.5 | -28.5 | 13.5 |
| Superior temporal gyrus, rolandic operculum, supramarginal gyrus, insula, postcentral gyrus, heschl gyrus | 1, 2, 13, 22, 40, 41, 42, 43 | L | 2679 | 7.70 | -49.5 | -33 | 19.5 |
| Precentral gyrus, postcentral gyrus, superior frontal gyrus, middle frontal gyrus | 2, 3, 4, 6, 40 | L | 1854 | 7.48 | -37.5 | -18 | 46.5 |
| Cerebellum crus2, cerebellum 7b, cerebellum 8, cerebellum crus1 | - | L | 1165 | 7.26 | -43.5 | -61.5 | -43.5 |
| Thalamus, hippocampus | 30 | L/R | 1113 | 7.93 | 0 | -18 | 1.5 |
| Inferior parietal lobule, postcentral gyrus, supramarginal gyrus, superior parietal lobule | 1, 2, 3, 7, 40 | L | 979 | 6.26 | -43.5 | -30 | 40.5 |
| Inferior frontal gyrus p. triangularis, middle frontal gyrus | 10, 45, 46 | L | 718 | 6.26 | -43.5 | 46.5 | 18 |
| Caudate nucleus, olfactory cortex | 25 | L | 578 | 7.01 | -10.5 | 12 | 13.5 |
| Precentral gyrus, inferior frontal gyrus p. opercularis, inferior frontal gyrus p. triangularis, middle frontal gyrus | 6, 9, 44 | L | 543 | 5.99 | -42 | 4.5 | 33 |
| Caudate nucleus | - | R | 440 | 6.61 | 9 | 21 | 4.5 |
| Anterior cingulate cortex, medial orbital gyrus, superior medial frontal gyrus | 10, 32 | L/R | 373 | 5.88 | 0 | 49.5 | -3 |
| Insula, inferior frontal gyrus p. triangularis | 13, 45, 47 | L | 361 | 6.44 | -33 | 24 | 6 |
| Postcentral gyrus, precentral gyrus | 4,6, 43 | L | 220 | 6.26 | -55.5 | -6 | 28.5 |
| Inferior frontal gyrus p. opercularis, precentral gyrus, inferior frontal gyrus p. triangularis | 9, 44, 45 | R | 211 | 5.48 | 52.5 | 9 | 18 |
| Middle frontal gyrus, superior frontal gyrus | 10 | L | 188 | 5.86 | -27 | 48 | 24 |
| Middle frontal gyrus, precentral gyrus, superior frontal gyrus | 6 | R | 176 | 5.77 | 31.5 | -3 | 52.5 |
| Middle frontal gyrus, superior frontal gyrus | 8 | L | 151 | 5.56 | -25.5 | 24 | 45 |
| Hippocampus | 28 | L | 133 | 5.67 | -21 | -13.5 | -10.5 |
| Postcentral gyrus, superior parietal lobule | 2, 3, 40 | L | 121 | 5.74 | -34.5 | -39 | 58.5 |
| Rolandic operculum, superior temporal gyrus, heschl gyrus | 6, 22, 42, 43 | L | 91 | 5.49 | -60 | -7.5 | 7.5 |
| Precentral gyrus | 6 | R | 66 | 5.47 | 42 | -12 | 51 |
| Superior temporal gyrus, insula | 13, 22 | L | 27 | 5.13 | -40.5 | -12 | -4.5 |
| Middle temporal gyrus | 21 | L | 23 | 5.15 | -57 | -10.5 | -12 |
| Putamen | - | L | 14 | 5.16 | -24 | 6 | 3 |
| **6 weeks >3 weeks** |  |  |  |  |  |  |  |
| Middle frontal gyrus, middle orbital gyrus, inferior frontal gyrus p. orbitalis, superior frontal gyrus, inferior frontal gyrus p. triangularis, superior frontal gyrus orbital part | 10, 11, 46, 47 | L | 1215 | 7.13 | -31.5 | 58.5 | 10.5 |
| Inferior frontal gyrus p. opercularis, precentral gyrus, inferior frontal gyrus p. triangularis | 8, 9, 44, 45 | L | 366 | 5.97 | -51 | 10.5 | 28.5 |
| Superior medial frontal gyrus | 6, 8, 9 | L/R | 321 | 5.79 | -3 | 36 | 42 |
| Middle frontal gyrus | 9, 10 | L | 298 | 6.59 | -28.5 | 46.5 | 28.5 |
| Inferior frontal gyrus p. triangularis, middle frontal gyrus | 10, 26 | L | 226 | 5.93 | -49.5 | 36 | 18 |
| Middle frontal gyrus, inferior frontal gyrus p. triangularis | 10, 46 | R | 162 | 5.26 | 46.5 | 42 | 12 |
| Superior frontal gyrus, middle frontal gyrus | 10 | R | 107 | 5.47 | 36 | 61.5 | 15 |
| Middle temporal gyrus | 21, 22 | L | 83 | 5.76 | -63 | -24 | -1.5 |
| Inferior frontal gyrus p. triangularis, inferior frontal gyrus p. opercularis | 44, 45 | L | 66 | 6.09 | -54 | 19.5 | 9 |
| Superior medial frontal gyrus, superior frontal gyrus | 10 | R | 55 | 5.41 | 13.5 | 70.5 | 10.5 |
| Middle frontal gyrus | 10 | R | 52 | 5.08 | 45 | 55.5 | 4.5 |
| Middle occipital gyrus, middle temporal gyrus | 19, 37 | L | 47 | 5.43 | -51 | -73.5 | 1.5 |
| Midcingulate cortex, paracentral lobule, precuneus | 5, 31 | R | 45 | 5.14 | 7.5 | -31.5 | 49.5 |
| Middle frontal gyrus, superior frontal gyrus | 8 | R | 43 | 5.16 | 28.5 | 18 | 54 |
| Medial orbital gyrus, gyrus rectus | 11 | L/R | 38 | 5.14 | -4.5 | 55.5 | -18 |
| Midcingulate cortex, paracentral lobule | - | L | 37 | 5.34 | -10.5 | -34.5 | 46.5 |
| Middle frontal gyrus | 9 | L | 29 | 5.28 | -43.5 | 21 | 37.5 |
| Supplementary motor area | 6 | L | 26 | 5.17 | -3 | 6 | 55.5 |
| Supramarginal gyrus, superior temporal gyrus | - | L | 26 | 5.13 | -54 | -39 | 24 |
| Middle frontal gyrus orbital part | 10 | R | 23 | 5.14 | 42 | 58.5 | -7.5 |
| Superior frontal gyrus, middle frontal gyrus | - | R | 22 | 5.11 | 30 | -1.5 | 61.5 |
| Superior medial frontal gyrus | 10 | L | 21 | 5.08 | -3 | 60 | 10.5 |
| Middle temporal gyrus | 21, 22 | L | 19 | 5.19 | -55.5 | -49.5 | 6 |
| Inferior parietal lobule | 40 | L | 15 | 5.10 | -52.5 | -30 | 46.5 |
| Middle frontal gyrus, superior frontal gyrus | 8 | R | 15 | 5.09 | 25.5 | 34.5 | 42 |
| Temporal pole mid, middle temporal gyrus | - | R | 16 | 5.14 | 57 | 6 | -19.5 |
| Superior frontal gyrus, superior orbital gyrus | 10 | R | 14 | 5.07 | 31.5 | 66 | 0 |
| Angular gyrus | - | L | 14 | 5.17 | -45 | -54 | 24 |
| Middle temporal gyrus | 22, 39 | L | 13 | 5.08 | -58.5 | -55.5 | 12 |
| Middle temporal gyrus | 21 | L | 12 | 5.09 | -64.5 | -13.5 | -19.5 |
| Gyrus rectus | 11 | L/R | 11 | 5.09 | 0 | 45 | -21 |
| Superior medial frontal gyrus | 9 | L | 11 | 5.00 | -1.5 | 52.5 | 22.5 |
| **9 weeks > 6 weeks (p < .001)** |  |  |  |  |  |  |  |
| Medial orbital gyrus, superior medial frontal gyrus, anterior cingulate cortex, superior frontal gyrus | 10, 11, 32 | L/R | 608 | 4.48 | -1.5 | 63 | -4.5 |
| **24 weeks > 12 weeks** |  |  |  |  |  |  |  |
| Middle frontal gyrus, superior frontal gyrus | 9, 10 | L | 197 | 5.96 | -31.5 | 46.5 | 36 |
| Supplementary motor area, superior medial frontal gyrus | 8, 32 | L/R | 75 | 5.28 | 1.5 | 22.5 | 48 |
| Superior medial frontal gyrus, midcingulate cortex | 9 | L/R | 42 | 5.16 | 1.5 | 43.5 | 30 |
| Pallidum | - | L | 41 | 5.68 | -16.5 | 1.5 | -4.5 |
| Pallidum | - | R | 12 | 5.44 | 13.5 | 1.5 | -6 |
| **24 weeks > 12 weeks (p < .001)** |  |  |  |  |  |  |  |
| Middle frontal gyrus, superior medial frontal gyrus, inferior frontal gyrus p. triangularis, superior frontal gyrus, supplementary motor area, midcingulate cortex, anterior cingulate cortex, precentral gyrus, inferior frontal gyrus p. opercularis, medial orbital gyrus, inferior frontal gyrus p. orbitalis, precuneus, paracentral lobule, insula, superior orbital gyrus | 4, 5, 6, 7, 8, 9, 10, 13, 24, 31, 32, 44, 45, 46, 47 | L/R | 15660 | 5.96 | -31.5 | 46.5 | 36 |
| Supramarginal gyrus middle temporal gyrus, angular gyrus, inferior parietal lobule, superior temporal gyrus, postcentral gyrus | 2, 13, 19, 22, 39, 40, 42 | L | 3553 | 4.67 | -64.5 | -42 | 28.5 |
| Middle frontal gyrus, superior frontal gyrus, inferior frontal gyrus p. opercularis, inferior frontal gyrus p. triangularis, precentral gyrus, superior medial frontal gyrus | 8, 9, 10, 46 | R | 2192 | 4.51 | 30 | 42 | 48 |
| Putamen, pallidum, insula, rolandic operculum, heschl gyrus, hippocampus | 13 | L | 1107 | 5.68 | -16.5 | 1.5 | -4.5 |
| Inferior temporal gyrus, middle temporal gyrus | 20, 37 | L | 720 | 4.15 | -58.5 | -39 | -24 |
| **childbirth > 24 weeks postpartum** |  |  |  |  |  |  |  |
| Caudate nucleus, putamen | - | L | 166 | 6.36 | -21 | 7.5 | 18 |
| Caudate nucleus | - | R | 41 | 5.48 | 21 | 10.5 | 15 |
| Voxels not found in atlases | - | - | 15 | 5.28 | 7.5 | -49.5 | -27 |
| **3 weeks > 24 weeks postpartum** |  |  |  |  |  |  |  |
| Thalamus | - | L | 67 | 5.8 | -21 | -18 | 15 |
| Thalamus | - | R | 66 | 6.08 | 19.5 | -18 | 16.5 |
| Voxels not found in atlases | - | - | 46 | 6.16 | 0 | -3 | -16.5 |
| Caudate, Putamen | - | L | 41 | 5.34 | -18 | 10.5 | 13.5 |

Note. p. = pars

Comparison between the postpartum and nulliparous brains

The results showing the smaller gray matter volume (GMV) in postpartum women compared to nulliparous women are depicted in Figure 5A (see Table S5).

Investigating t-contrasts revealed significantly smaller GMVs in postpartum women compared to nulliparous women. Within the first 10 days after childbirth, differences were detected in the bilateral striatum, pallidum, amygdala, hippocampus, parahippocampal cortex, insular cortex, midcingulate cortex and left anterior cingulate cortex. Further, smaller volume was detected in the bilateral fusiform gyrus, lingual gyrus, olfactory cortex, the orbital part of the frontal cortex, the middle and superior frontal gyri and superior medial frontal gyrus, the paracentral lobule, supplementary motor area, the angular gyrus, the precuneus, the superior parietal lobule, the temporal cortex and superior pole, and the cerebellum, as well as in the left postcentral gyrus, supramarginal gyrus, inferior parietal lobule and gyrus rectus, and the right occipital cortex. At 3 weeks postpartum, smaller GMV was detected in mothers in the bilateral amygdala, hippocampus, parahippocampal gyrus, midcingulate cortex, pallidum, putamen, superior medial frontal gyrus, supplementary motor area, and the superior parietal lobule, as well as the left fusiform gyrus, inferior and middle temporal gyri, and cerebellum, and the right superior and middle frontal gyri, angular gyrus, and middle and superior occipital gyri. At 6 weeks postpartum, compared to nulliparous women, smaller volume was detected in the bilateral amygdala, hippocampus, parahippocampal gyrus, putamen, superior medial frontal gyrus, and supplementary motor area, as well as the left pallidum, fusiform gyrus, middle temporal gyrus, and cerebellum, and the right midcingulate cortex, superior parietal lobule, and the middle and superior occipital gyri of postpartum women. At 9 weeks postpartum, smaller GMV was detected in postpartum women in the bilateral supplementary motor area, the left amygdala, hippocampus, putamen, lingual gyrus, and middle temporal gyrus, and the right superior parietal and occipital gyri.

Table S5*.* Brain Regions showing larger gray matter volume in nulliparous women than postpartum women (t-contrast from random-effects GLM, *p* < .05, cluster-level FWE correction; size ≥ 10 voxels).

| **Anatomical Region** | **Brodmann** | **Side** | **size** | **T** | **x** | **y** | **z** |
| --- | --- | --- | --- | --- | --- | --- | --- |
| **nulliparous > postpartum childbirth** |  |  |  |  |  |  |  |
| Cerebellum 6, crus 1, fusiform gyrus, crus 2, lingual gyrus, cerebellum 4, 5, 8, inferior temporal gyrus, vermis 7, cerebellum 7b | 19, 20, 37 | L | 3233 | 7.16 | -40.5 | -54 | -22.5 |
| Hippocampus, amygdala, parahippocampal gyrus gyrus, putamen, superior temporal pole, olfactory cortex, pallidum, fusiform gyrus, insula, caudate nucleus | 28, 34, 35, 36, 37, 38, | L | 2410 | 8.78 | -22.5 | -3 | -12 |
| Midcingulate cortex, supplementary motor area, superior medial frontal gyrus, supplementary motor area, paracentral lobule, superior frontal gyrus | 5, 6, 7, 8, 9, 24, 31, 32 | L/R | 2307 | 8.44 | 3 | 13.5 | 46.5 |
| Amygdala, hippocampus, parahippocampal gyrus cortex, putamen, pallidum, olfactory cortex, superior temporal pole, caudate nucleus, insula | 28, 34, 47 | R | 1584 | 7.55 | 18 | 0 | -13.5 |
| Cerebellum 6, crus 1, fusiform gyrus | 19, 37 | R | 1155 | 6.40 | 31.5 | 66 | -25.5 |
| Middle temporal gyrus, superior temporal gyrus | 21, 22 | L | 416 | 6.89 | -66 | -19.5 | -6 |
| Superior occipital gyrus, superior parietal lobule, middle occipital gyrus, angular gyrus, precuneus | 7, 19 | R | 328 | 7.15 | 22.5 | -63 | 48 |
| Midcingulate cortex, paracentral lobule | 31 | R | 150 | 7.02 | 10.5 | -36 | 43.5 |
| Olfactory cortex, gyrus rectus, medial orbital gyrus | 11, 25 | L | 146 | 5.87 | -3 | 15 | -16.5 |
| Superior medial frontal gyrus, anterior cingulate cortex | 10 | L | 109 | 6.53 | -9 | 51 | 6 |
| Middle frontal gyrus, superior frontal, superior orbital gyrus, middle orbital gyrus | 10 | R | 99 | 6.46 | 31.5 | 60 | 1.5 |
| Postcentral gyrus, inferior parietal lobule | 2, 3, 40 | L | 98 | 6.04 | -42 | -25.5 | 43.5 |
| Middle temporal gyrus, superior temporal gyrus, angular gyrus, supramarginal gyrus | 22, 39, 40 | L | 92 | 6.12 | -58.5 | -57 | 21 |
| Midcingulate cortex, superior medial frontal gyrus | 9, 32 | L | 62 | 5.93 | -9 | 25.5 | 34.5 |
| Middle temporal gyrus | - | L | 58 | 5.77 | -51 | -18 | -16.5 |
| Superior orbital gyrus | 10 | L | 44 | 5.71 | -24 | 63 | -3 |
| Middle occipital gyrus | 19, 39 | R | 51 | 5.92 | 37.5 | -81 | 28.5 |
| Superior parietal lobule, precuneus | 7 | L | 42 | 6.17 | -16.5 | -64.5 | 49.5 |
| Middle occipital gyrus | 18, 19 | R | 33 | 5.55 | 31.5 | -88.5 | 16.5 |
| Middle temporal gyrus | 21 | L | 19 | 5.83 | -58.5 | -25.5 | -13.5 |
| Crus 2 | - | R | 15 | 5.49 | 40.5 | -70.5 | -43.5 |
| Middle temporal gyrus, superior temporal gyrus | 21, 22 | R | 14 | 5.49 | 61.5 | -27 | -3 |
| Middle frontal gyrus | 10, 46 | R | 11 | 5.50 | 43.5 | 40.5 | 24 |
| **nulliparous > postpartum 3 weeks** |  |  |  |  |  |  |  |
| Amygdala, hippocampus, putamen, pallidum parahippocampal gyrus cortex | 28, 34- | L/R | 639 | 6.88 | -27 | -9 | -10.5 |
| Supplementary motor area, superior medial frontal gyrus, middle cingulate cortex | 6, 8, 32 | L/R | 628 | 7.70 | 3 | 13.5 | 46.5 |
| Amygdala, hippocampus, parahippocampal gyrus cortex, putamen, pallidum | 34 | R | 472 | 6.50 | 18 | -1.5 | -10.5 |
| Middle cingulate cortex | 24, 31 | L | 92 | 6.18 | -6 | -21 | 45 |
| Middle temporal gyrus | 21 | L | 82 | 6.36 | -67.5 | -19.5 | -4.5 |
| Fusiform gyrus, cerebellum 6 | 37 | L | 62 | 6.01 | -40.5 | -54 | -22.5 |
| Superior occipital gyrus, superior parietal lobule, angular gyrus | 7 | R | 55 | 6.38 | 22.5 | -64.5 | 49.5 |
| Superior occipital gyrus, middle occipital gyrus | 19 | R | 36 | 5.84 | 31.5 | -73.5 | 42 |
| Middle cingulate cortex | - | R | 22 | 5.93 | 10.5 | -36 | 42 |
| Superior medial frontal gyrus | 10 | L | 21 | 5.87 | -7.5 | 51 | 6 |
| Cerebellum 6 | - | L | 10 | 5.39 | -28.5 | -72 | -24 |
| **nulliparous > postpartum 6 weeks** |  |  |  |  |  |  |  |
| Amygdala, hippocampus, putamen, pallidum | 34 | L | 522 | 6.96 | -25.5 | -9 | -9 |
| Supplementary motor area, middle cingulate cortex, superior medial frontal gyrus | 6, 8, 32 | L/R | 241 | 6.81 | 3 | 13.5 | 46.5 |
| Amygdala, parahippocampal gyrus gyrus, hippocampus | 34 | R | 97 | 5.71 | 19.5 | -1.5 | -10.5 |
| Putamen, hippocampus, amygdala | - | R | 80 | 6.11 | 31.5 | -18 | -4.5 |
| fusiform gyrus, cerebellum 6 | 37 | L | 27 | 5.72 | -40.5 | -54 | -22.5 |
| Voxel not found in atlases | - |  | 26 | 6.19 | 9 | 1.5 | -6 |
| Superior occipital gyrus, superior parietal lobule | 7 | R | 23 | 5.81 | 22.5 | -64.5 | 49.5 |
| Middle temporal gyrus | 21 | L | 19 | 5.81 | -67.5 | -19.5 | -4.5 |
| Parahippocampal gyrus gyrus | 35 | L | 17 | 5.51 | -19.5 | -24 | -15 |
| **nulliparous > postpartum 9 weeks** |  |  |  |  |  |  |  |
| Amygdala, hippocampus, putamen | - | L | 458 | 6.86 | -24 | -6 | -12 |
| Supplementary motor area | 6, 32 | L/R | 71 | 6.06 | 4.5 | 12 | 46.5 |
| Superior occipital gyrus, superior parietal lobule | 7 | R | 13 | 5.63 | 21 | -63 | 48 |
| **nulliparous > postpartum 12 weeks** |  |  |  |  |  |  |  |
| Amygdala, hippocampus, putamen | - | L | 301 | 6.32 | -24 | -6 | -12 |
| Supplementary motor area | 6, 8, 32 | L/R | 105 | 6.32 | 4.5 | 12 | 48 |
| Hippocampus, amygdala | - | R | 15 | 5.59 | 30 | -10.5 | -10.5 |
| Superior occipital gyrus, superior parietal lobule | 7 | R | 11 | 5.61 | 22.5 | -64.5 | 49.5 |
| **nulliparous > postpartum 24 weeks** |  |  |  |  |  |  |  |
| Amygdala, putamen, hippocampus | - | L | 108 | 6.00 | -25.5 | -4.5 | -13.5 |
| Supplementary motor area | 6, 32 | R | 37 | 5.80 | 4.5 | 12 | 48 |

*Figure S1.*

Table S6. Intercorrelations among receptor maps.

|  | NR3C1 | NR3C2 | ESR1 | PGR | OXTR | GABA | mGluR5 |
| --- | --- | --- | --- | --- | --- | --- | --- |
| NR3C1 |  |  |  |  |  |  |  |
| NR3C2 | .96 |  |  |  |  |  |  |
| ESR1 | .89 | .88 |  |  |  |  |  |
| PGR | .74 | .77 | .82 |  |  |  |  |
| OXTR | .70 | .75 | .76 | .89 |  |  |  |
| GABA | .36 | .37 | .04 | -.01 | .08 |  |  |
| mGluR5 | .26 | .31 | .02 | .28 | .40 | .62 |  |

Note. NR3C1/NR3C2 = nuclear receptor subfamily 3, group C, member 1 and 2, respectively; ESR1 = Estrogen Receptor 1; PGR = progesterone receptor; OXTR = oxytocin receptor; GABA_A_ = GABA_A_ receptor; mGluR5 = metabotropic glutamate receptor 5.

Table S7. Receptor Colocalizations with brain volumetric changes in postpartum women.

|  | **t0<t1** | | | **t1<t2** | | | **t2<t3** | | | **t4<t5** | | |
| --- | --- | --- | --- | --- | --- | --- | --- | --- | --- | --- | --- | --- |
|  | **spearman rho** | **p** | **Q** | **spearman rho** | **p** | **q** | **spearman rho** | **p** | **q** | **spearman rho** | **p** | **q** |
| NR3C1 | .52 | <.001 | .007* | .34 | <.001 | .014* | -.03 | .766 | .050 | .19 | .042 | .029 |
| NR3C2 | .58 | <.001 | .014* | .30 | <.001 | .029* | -.08 | .357 | .036 | .14 | .137 | .036 |
| ESR1 | .26 | .005 | .036* | -.02 | .849 | .050 | -.43 | <.001 | .007* | -.13 | .173 | .043 |
| PGR | .22 | .016 | .043* | .05 | .607 | .043 | -.10 | .292 | .029 | -.02 | .813 | .050 |
| OXTR | .03 | .732 | .050 | .16 | .076 | .036 | .06 | .528 | .043 | .24 | .010 | .021* |
| GABA_A_ | .75 | <.001 | .021* | .36 | <.001 | .007* | .65 | <.001 | .021* | .35 | <.001 | .007* |
| mGluR5 | .62 | <.001 | .029* | .54 | <.001 | .021* | .60 | <.001 | .014* | .58 | <.001 | .014* |

Note. reported *p* values are uncorrected. *q* values are FDR corrected; significant corrected results are marked with * (*p* <.05). t0 = within 10 days of childbirth; t1 = 3 weeks postpartum (pp.); t2 = 6 weeks pp.; t3 = 9 weeks pp.; t4 = 12 weeks pp.; t5 = 24 weeks pp.; NR3C1/NR3C2 = nuclear receptor subfamily 3, group C, member 1 and 2, respectively; ESR1 = Estrogen Receptor 1; PGR = progesterone receptor; OXTR = oxytocin receptor; GABA_A_ = GABA_A_ receptor; mGluR5 = metabotropic glutamate receptor 5.

Table S8. Receptor Colocalizations with brain differences between nulliparous and postpartum women at all time points.

|  | **Within 10 days of childbirth** | | | **3 weeks pp.** | | | **6 weeks pp.** | | | **9 weeks pp.** | | | **12 weeks pp.** | | | **24 weeks pp.** | | |
| --- | --- | --- | --- | --- | --- | --- | --- | --- | --- | --- | --- | --- | --- | --- | --- | --- | --- | --- |
|  | **spearman rho** | **p** | **q** | **spearman rho** | **p** | **q** | **spearman rho** | **p** | **q** | **spearman rho** | **p** | **q** | **spearman rho** | **p** | **q** | **spearman rho** | **p** | **q** |
| NR3C1 | -.20 | .030 | .007 | -.27 | .003 | .007* | -.39 | <.001 | .007* | -.33 | <.001 | .014* | -.34 | <.001 | .014* | -.32 | <.001 | .007* |
| NR3C2 | -.17 | .068 | .014 | -.23 | .014 | .014* | -.34 | <.001 | .014* | -.28 | .002 | .021* | -.28 | .002 | .021* | -.26 | .005 | .021* |
| ESR1 | -.12 | .208 | .036 | -.20 | .035 | .029 | -.21 | .024 | .029* | -.15 | .115 | .029 | -.16 | .092 | .036 | -.17 | .069 | .029 |
| PGR | -.10 | .270 | .043 | -.15 | .100 | .036 | -.11 | .223 | .043 | -.09 | .333 | .050 | -.09 | .337 | .050 | -.10 | .309 | .050 |
| OXTR | -.13 | .167 | .029 | -.07 | .432 | .043 | -.10 | .275 | .050 | -.10 | .291 | .043 | -.09 | .322 | .043 | -.15 | .104 | .043 |
| GABA_A_ | -.13 | .166 | .021 | -.21 | .023 | .021 | -.29 | .002 | .021* | -.32 | <.001 | .007* | -.33 | <.001 | .007* | -.29 | .001 | .014* |
| mGluR5 | -.03 | .827 | .050 | -.08 | .561 | .050 | -.16 | .223 | .036 | -.20 | .128 | .036 | -.23 | .078 | .029 | -.23 | .074 | .036 |

Note. reported *p* values are uncorrected. *q* values are FDR corrected; significant corrected results are marked with * (*p* <.05). pp. = postpartum; NR3C1/NR3C2 = nuclear receptor subfamily 3, group C, member 1 and 2, respectively; ESR1 = Estrogen Receptor 1; PGR = progesterone receptor; OXTR = oxytocin receptor; GABA_A_ = GABA_A_ receptor; mGluR5 = metabotropic glutamate receptor 5.

Table S9. Specificity of significant associations between receptor maps and brain volumetric changes in postpartum women. Multiple linear regression models including the receptor maps that showed significant associations with maternal brain changes between postpartum time points.

|  | **t0<t1** | | | **t1<t2** | | | **t2<t3** | | | **t4<t5** | | |
| --- | --- | --- | --- | --- | --- | --- | --- | --- | --- | --- | --- | --- |
|  | **Mean Beta** | **p** | **q*** | **Mean Beta** | **p** | **q*** | **Mean Beta** | **p** | **q*** | **Mean Beta** | **p** | **q*** |
| NR3C1 | 0.21 | .367 | .033 | 0.87 | <.001 | .013* |  |  |  |  |  |  |
| NR3C2 | 0.91 | <.001 | .008* | -0.73 | <.001 | .025* |  |  |  |  |  |  |
| ESR1 | -0.27 | .651 | .05 |  |  |  | -0.43 | <.001 | .017* |  |  |  |
| PGR | -1.65 | <.001 | .017* |  |  |  |  |  |  |  |  |  |
| OXTR |  |  |  |  |  |  |  |  |  | -0.04 | .729 | .05 |
| GABA | 0.01 | .551 | .042 | 0.01 | .593 | .05 | 0.01 | .007 | .05* | 0.01 | .593 | .033 |
| mGluR5 | 0.03 | .029 | .025 | 0.02 | .003 | .038* | 0.02 | .004 | .033* | 0.02 | .003 | .017* |

Note. Cells of receptor maps with no significant association with the brain volume at the respective timepoint are left empty. Reported *p* values are uncorrected. *q* values are FDR corrected; significant corrected results are marked with * (*p* <.05). t0 = within 10 days of childbirth; t1 = 3 weeks postpartum (pp.); t2 = 6 weeks pp.; t3 = 9 weeks pp.; t4 = 12 weeks pp.; t5 = 24 weeks pp.; NR3C1/NR3C2 = nuclear receptor subfamily 3, group C, member 1 and 2, respectively; ESR1 = Estrogen Receptor 1; PGR = progesterone receptor; OXTR = oxytocin receptor; GABA_A_ = GABA_A_ receptor; mGluR5 = metabotropic glutamate receptor 5.

Table S10. Specificity of significant associations between receptor maps and brain differences between nulliparous and postpartum women per timepoint. Multiple linear regression models including the receptor maps that showed significant associations with differences between maternal and nulliparous brains.

|  | **3 weeks pp.** | | | **6 weeks pp.** | | | **9 weeks pp.** | | | **12 weeks pp.** | | | **24 weeks pp.** | | |
| --- | --- | --- | --- | --- | --- | --- | --- | --- | --- | --- | --- | --- | --- | --- | --- |
|  | **Mean Beta** | **p** | **q*** | **Mean Beta** | **p** | **q*** | **Mean Beta** | **p** | **q*** | **Mean Beta** | **p** | **q*** | **Mean Beta** | **p** | **q*** |
| NR3C1 | -0.51 | <.001 | .025* | -0.88 | <.001 | .013* | -0.62 | <.001 | .017* | -0.67 | <.001 | .017* | -0.66 | <.001 | .017* |
| NR3C2 | 0.41 | <.001 | .05* | 0.17 | .045 | .038 | 0.67 | <.001 | .033* | 0.72 | <.001 | .033* | 0.71 | <.001 | .033* |
| ESR1 |  |  |  | 1.17 | .002 | .025* |  |  |  |  |  |  |  |  |  |
| GABA |  |  |  | <0.01 | .661 | .05 | -0.02 | .003 | .05* | -0.02 | .003 | .05* | -0.02 | .004 | .05* |

Note. Cells for receptor maps with no significant association with the brain volume at the respective timepoint are left empty. Reported *p* values are uncorrected. *q* values are FDR corrected; significant corrected results are marked with * (*p* <.05). pp. = postpartum; NR3C1/NR3C2 = nuclear receptor subfamily 3, group C, member 1 and 2, respectively; ESR1 = Estrogen Receptor 1; PGR = progesterone receptor; OXTR = oxytocin receptor; GABA_A_ = GABA_A_ receptor; mGluR5 = metabotropic glutamate receptor 5.

Table S11. Control analyses of correlations between maternal GMV changes and Beta-2 Adrenergic (ADRB2), Serotonin 1A (HTR1A) and Dopamine (D2) receptor distributions.

|  | **t0<t1** | | | **t1<t2** | | | **t2<t3** | | | **t4<t5** | | |
| --- | --- | --- | --- | --- | --- | --- | --- | --- | --- | --- | --- | --- |
|  | **spearman rho** | **p** | **q*** | **spearman rho** | **p** | **q*** | **spearman rho** | **p** | **q*** | **spearman rho** | **p** | **q*** |
| 5HT1A | -.09 | .468 | .514 | .29 | .026 | .078 | .15 | .222 | .333 | .07 | .514 | .514 |
| ADBR2 | .10 | .488 | .514 | .39 | .013 | .052 | .10 | .475 | .514 | .22 | .063 | .126 |
| D1 | -.23 | <.010 | .052 | -.24 | .007 | .052 | .18 | .059 | .126 | -.15 | .105 | .180 |

Note. reported *p* values are uncorrected. *q* values are FDR corrected; significant corrected results are marked with * (*p* <.05). t0 = within 10 days of childbirth; t1 = 3 weeks postpartum (pp.); t2 = 6 weeks pp.; t3 = 9 weeks pp.; t4 = 12 weeks pp.; t5 = 24 weeks pp.; 5HT1A = Serotonin 1A receptor, ADBR2 = beta‑2 adrenergic receptor, D1 = Dopamine receptor.

Table S12. Brain regions (GMV) showing associations with hormonal and behavioral measures in postpartum women (multiple linear regression, cluster-forming threshold *p* < .001 uncorrected, cluster-level FWE-corrected *p* < .05).

| **timepoint** | **parameter** | **direction** | **Anatomical Region** | **Brodmann** | **Side** | **Size** | **T** | **x** | **y** | **z** |
| --- | --- | --- | --- | --- | --- | --- | --- | --- | --- | --- |
| 6 weeks pp. | Estradiol | positive | Cerebellum 6, crus 1, vermis 6, 7, lingual gyrus | 18 | L | 686 | 5.22 | -4.5 | -75 | -18 |
| 12 weeks pp. | MPAS QoA | positive | Middle temporal gyrus, middle occipital gyrus, angular gyrus | 19, 39 | R | 787 | 5.74 | 46.5 | -69 | 19.5 |
|  |  | positive | Inferior frontal gyrus p. opercularis, precentral gyrus, inferior frontal gyrus p. triangularis, middle frontal gyrus | 6, 9, 44 | L | 697 | 6.07 | -43.5 | 10.5 | 25.5 |
|  | MPAS PiI | negative | Crus 1, 2, cerebellum 7b | - | L | 892 | 5.62 | -55.5 | -61.5 | -45 |
| 24 weeks pp. | MPAS PiI | negative | Crus 1, 2, cerebellum 7b | - | L | 656 | 5.64 | -51 | -43.5 | -43.5 |
|  | MPAS AoH | negative | Hippocampus, parahippocampal gyrus, amygdala | 28, 34, 35 | L | 617 | 6.53 | -16.5 | -16.5 | -19.5 |

Note. pp. = postpartum, MPAS = maternal postnatal attachment scale, QoA = quality of attachment, PiI = pleasure in interaction, AoH = absence of hostility.

*Figure S2.*

**MRI results of surface-based analyses**

Development of maternal cortical thickness and sulcus depth throughout the postpartum period

Throughout the postpartum period increases in cortical thickness were observed mainly from childbirth to 3 weeks postpartum in the bilateral frontal cortex and orbitofrontal cortex, parietal, occipital and temporal cortices, insula and cingulate cortex. When adjusting the threshold to *p* < .001 uncorrected, significant increases from 3 to 6 weeks postpartum could be detected in the left middle frontal gyrus as well as the right frontal gyri, middle and superior temporal gyri, inferior parietal lobule, supramarginal gyrus and precentral gyrus (see Figure S2A and Table S13).

Changes in sulcus depth in postpartum brains indicate an increase across the time points, most prominently from childbirth to 3 weeks postpartum in the bilateral frontal, parietal and temporal cortices. From 3 to 6 weeks the left inferior frontal gyrus and lateral orbitofrontal cortex, right middle frontal gyrus and inferior parietal cortex, and left insula showed an increase in sulcus depth. From 6 to 9 weeks postpartum the left inferior frontal and precentral gyri as well as the right superior temporal gyrus were affected. No increase was detected from 9 to 12 weeks or 12 to 24 weeks, but from 9 to 24 weeks postpartum in the left paracentral lobule and precuneus (see Figure S3 and Table S14).

Table S13. Brain regions showing increase in cortical thickness in postpartum women across the postpartum period (t-contrast from random-effects GLM, *p* < .05, cluster-level FWE correction, size ≥ 10 voxels).

| **Anatomical Region** | **Side** | **Size** | **Value** |
| --- | --- | --- | --- |
| **3 weeks postpartum > childbirth** |  |  |  |
| Superior parietal gyrus, inferior parietal gyrus, lateral occipital cortex | L | 433 | 6.5 |
| Supramarginal gyrus, Insula, postcentral gyrus, superior temporal gyrus, transverse temporal gyrus | L | 287 | 6.4 |
| Lateral occipital cortex, inferior temporal gyrus, fusiform gyrus | L | 239 | 5.5 |
| Precuneus cortex, cuneus cortex, superior parietal cortex | L | 201 | 5.2 |
| Inferior parietal cortex, lateral occipital cortex | L | 170 | 5.5 |
| Postcentral gyrus, supramarginal gyrus | L | 149 | 6.2 |
| Rostral middle frontal gyrus | L | 123 | 5.2 |
| Caudal middle frontal gyrus, precentral gyrus, Rostral middle frontal gyrus | L | 116 | 5.6 |
| Inferior frontal gyrus p. triangularis, Lateral orbitofrontal gyrus, Inferior frontal gyrus p. opercularis | L | 103 | 5.2 |
| Rostral middle frontal gyrus | L | 76 | 5.2 |
| Superior frontal gyrus | L | 75 | 5.3 |
| Fusiform gyrus, lingual gyrus, lateral occipital cortex | L | 75 | 4.8 |
| Superior frontal gyrus, paracentral lobule, posterior cingulate cortex | L | 70 | 5.2 |
| Isthmus cingulate cortex, precuneus cortex, posterior cingulate cortex | L | 68 | 5.7 |
| Postcentral gyrus, precentral gyrus | L | 55 | 5.0 |
| Superior parietal cortex, postcentral gyrus | L | 32 | 4.7 |
| Precentral gyrus, superior frontal gyrus | L | 25 | 4.9 |
| Superior frontal gyrus | L | 22 | 4.8 |
| Precentral gyrus | L | 22 | 4.9 |
| Precentral gyrus | L | 21 | 4.7 |
| Banks of the superior temporal sulcus | L | 17 | 4.7 |
| Precentral gyrus | L | 11 | 4.8 |
| Inferior frontal gyrus p. opercularis | L | 11 | 4.7 |
| Precuneus cortex | L | 10 | 4.6 |
| Supramarginal gyrus, postcentral gyrus, superior parietal cortex | R | 262 | 5.4 |
| Paracentral lobule, superior frontal gyrus | R | 196 | 6.2 |
| Supramarginal gyrus, superior temporal gyrus | R | 110 | 5.3 |
| Superior parietal cortex | R | 97 | 5.6 |
| Precuneus cortex | R | 94 | 6.15 |
| Cuneus cortex, precuneus cortex | R | 82 | 5.5 |
| Posterior cingulate cortex | R | 34 | 4.9 |
| Precuneus cortex, paracentral lobule | R | 33 | 4.9 |
| Superior frontal gyrus | R | 27 | 5.1 |
| Superior parietal cortex, cuneus cortex | R | 22 | 4.6 |
| Precentral gyrus | R | 21 | 4.7 |
| Lateral orbitofrontal gyrus | R | 21 | 5.0 |
| Inferior parietal cortex | R | 15 | 4.8 |
| Precentral gyrus | R | 15 | 4.6 |
| Lateral occipital cortex | R | 12 | 4.7 |
| Inferior frontal gyrus p. triangularis | R | 10 | 4.6 |
| Postcentral gyrus | R | 10 | 4.5 |
| **6 weeks > 3 weeks postpartum (p < .001)** |  |  |  |
| Rostral middle frontal gyrus, Caudal middle frontal gyrus | L | 250 | 4.4 |
| Rostral middle frontal gyrus | L | 105 | 4.1 |
| Superior frontal gyrus, Caudal middle frontal gyrus, precentral gyrus | R | 460 | 4.4 |
| Banks of the superior temporal sulcus, Superior temporal gyrus, Inferior parietal cortex, Middle temporal gyrus, supramarginal gyrus | R | 237 | 4.1 |
| Superior frontal gyrus | R | 144 | 4.1 |
| Rostral middle frontal gyrus, inferior frontal gyrus p. triangularis | R | 87 | 3.9 |
| **24 > 12 weeks postpartum (p < .001)** |  |  |  |
| Superior frontal gyrus | L | 99 | 4.2 |

Note. P. = pars.

Table S14. Brain Regions showing increase in sulcus depth in postpartum women across the postpartum period (t-contrast from random-effects GLM, *p* < .05, cluster-level FWE correction).

| **Anatomical Region** | **Side** | **Size** | **Value** |
| --- | --- | --- | --- |
| **3 weeks postpartum > childbirth** |  |  |  |
| Precentral gyrus, Caudal middle frontal gyrus | L | 203 | 5.4 |
| Rostral middle frontal gyrus | L | 143 | 4.8 |
| Superior temporal gyrus, transverse temporal gyrus | L | 131 | 5.5 |
| Rostral middle frontal gyrus, inferior frontal gyrus p. triangularis | L | 129 | 5.2 |
| Postcentral gyrus, supramarginal gyrus | L | 27 | 4.4 |
| Precentral gyrus | L | 21 | 4.4 |
| Inferior parietal gyrus, supramarginal gyrus | L | 17 | 4.5 |
| Precentral gyrus, postcentral gyrus | R | 294 | 5.0 |
| Supramarginal, Postcentral gyrus, superior parietal gyrus | R | 294 | 5.0 |
| Banks of the superior temporal sulcus, Middle temporal gyrus, superior temporal gyrus | R | 122 | 5.1 |
| Superior parietal gyrus, inferior parietal gyrus | R | 43 | 4.6 |
| Transverse temporal gyrus | R | 29 | 4.9 |
| Caudal middle frontal gyrus | R | 23 | 4.6 |
| **6 weeks > 3 weeks postpartum** |  |  |  |
| Inferior frontal gyrus p. triangularis, lateral orbitofrontal cortex, insula, inferior frontal gyrus p. opercularis, inferior frontal gyrus p. orbitalis | L | 239 | 5.4 |
| Inferior parietal gyrus, supramarginal gyrus | R | 18 | 4.5 |
| Rostral middle frontal gyrus | R | 10 | 4.3 |
| **9 weeks > 6 weeks postpartum** |  |  |  |
| Precentral gyrus, inferior frontal gyrus p. opercularis | L | 36 | 4.6 |
| Superior temporal gyrus | R | 10 | 4.4 |
| **24 weeks > 9 weeks postpartum** |  |  |  |
| Paracentral lobule, precuneus cortex | L | 16 | 4.4 |

Note. P. = pars.

Differences between nulliparous and postpartum brains in cortical thickness

When comparing nulliparous to postpartum brains, larger cortical thickness was detected in the nulliparous brains most prominently at childbirth in the frontal, parietal, occipital and temporal cortices, the insula, and cingulate cortex. These differences diminish over the following time points. After 3 weeks postpartum, differences remain in the frontal, parietal and temporal cortices. After 6 weeks postpartum, significant differences could only be detected using an uncorrected cluster-forming threshold *p* < .001, namely in the frontal, parietal and temporal cortices, and the insula and anterior cingulate cortex (ACC). After 9 weeks, the differences remain significant in the frontal, parietal and temporal cortices and the ACC. At 12 weeks, results revealed smaller cortical thickness in postpartum women in the bilateral superior temporal lobe, the left medial orbitofrontal gyrus, superior parietal lobule, and anterior cingulate cortex, as well as the right inferior parietal lobule (see Table S15).

At 24 weeks postpartum, reductions persisted in the bilateral inferior parietal lobule, superior temporal lobe, and left superior parietal lobule (see Figure S2B and Table S15).

Table S15. Brain regions showing larger cortical thickness in nulliparous women than postpartum women (t-contrast from random-effects GLM, *p* < .05, cluster-level FWE correction, size ≥ 10 voxels).

| **Anatomical Region** | **Side** | **Size** | **Value** |
| --- | --- | --- | --- |
| **nulliparous > childbirth** |  |  |  |
| Superior parietal gyrus, inferior parietal gyrus | L | 275 | 5.7 |
| Precuneus cortex | L | 162 | 6.6 |
| Rostral middle frontal gyrus | L | 155 | 6.0 |
| Superior temporal gyrus, transverse temporal gyrus | L | 141 | 5.7 |
| Superior frontal gyrus, caudal middle frontal gyrus | L | 134 | 6.1 |
| Superior frontal gyrus | L | 79 | 5.2 |
| Paracentral lobule | L | 67 | 6.3 |
| Superior parietal gyrus | L | 54 | 5.7 |
| Precuneus cortex, cuneus cortex | L | 53 | 5.6 |
| Superior parietal gyrus, inferior parietal gyrus | L | 50 | 5.8 |
| Rostral middle frontal gyrus, caudal middle frontal gyrus | L | 45 | 5.4 |
| Supramarginal gyrus | L | 44 | 5.2 |
| Inferior parietal gyrus | L | 43 | 5.3 |
| Medial orbitofrontal gyrus | L | 40 | 5.4 |
| Precentral gyrus, postcentral gyrus, insula | L | 35 | 5.6 |
| Caudal middle frontal gyrus | L | 32 | 5.5 |
| Supramarginal gyrus | L | 27 | 5.5 |
| Inferior frontal gyrus p. triangularis | L | 26 | 5.1 |
| Banks of the superior temporal sulcus | L | 14 | 5.1 |
| Anterior cingulate cortex caudal division, superior frontal gyrus | L | 12 | 5.0 |
| Rostral middle frontal gyrus, caudal middle frontal gyrus | R | 245 | 6.0 |
| Precuneus cortex, superior parietal gyrus | R | 177 | 6.7 |
| Inferior parietal gyrus, supramarginal gyrus, banks of the superior temporal sulcus | R | 120 | 5.7 |
| Rostral middle frontal gyrus | R | 61 | 5.2 |
| Superior frontal gyrus, rostral middle frontal gyrus | R | 52 | 5.2 |
| Inferior parietal gyrus, supramarginal gyrus | R | 45 | 5.9 |
| Superior frontal gyrus, medial orbitofrontal gyrus, frontal pole | R | 39 | 5.2 |
| Inferior parietal gyrus | R | 37 | 5.6 |
| Superior parietal gyrus | R | 28 | 5.2 |
| Rostral middle frontal gyrus, inferior frontal gyrus p. orbitalis | R | 25 | 5.6 |
| Precentral gyrus | R | 21 | 5.3 |
| Superior frontal gyrus | R | 18 | 5.0 |
| Superior frontal gyrus | R | 15 | 5.0 |
| Paracentral lobule | R | 14 | 5.1 |
| Superior parietal gyrus | R | 14 | 5.1 |
| **Nulliparous > 3 weeks postpartum** |  |  |  |
| Medial orbitofrontal gyrus | L | 13 | 5.2 |
| Paracentral lobule | L | 11 | 5.1 |
| Supramarginal gyrus, inferior parietal gyrus, banks of the superior temporal sulcus | R | 66 | 5.3 |
| Inferior parietal gyrus, supramarginal gyrus | R | 19 | 5.4 |
| Rostral middle frontal gyrus | R | 15 | 5.2 |
| **Nulliparous > 6 weeks postpartum (p < .001)** |  |  |  |
| Superior parietal gyrus, inferior parietal gyrus | L | 291 | 4.1 |
| Banks of the superior temporal sulcus, superior temporal gyrus, middle temporal gyrus | L | 280 | 4.2 |
| Superior temporal gyrus, transverse temporal gyrus | L | 263 | 4.4 |
| Medial orbitofrontal gyrus, anterior cingulate cortex rostral division | L | 160 | 4.7 |
| Insula, precentral gyrus, postcentral gyrus | L | 123 | 4.5 |
| Inferior parietal gyrus, supramarginal gyrus, banks of the superior temporal sulcus | R | 254 | 4.7 |
| Rostral middle frontal gyrus, caudal middle frontal gyrus | R | 183 | 4.1 |
| Middle temporal gyrus, superior temporal gyrus | R | 130 | 3.8 |
| Precuneus cortex, superior parietal gyrus | R | 97 | 4.1 |
| **Nulliparous > 9 weeks postpartum (p < .001)** |  |  |  |
| Superior temporal gyrus, transverse temporal gyrus | L | 291 | 4.2 |
| Superior parietal gyrus, inferior parietal gyrus | L | 189 | 3.9 |
| Superior temporal gyrus, banks of the superior temporal sulcus, middle temporal gyrus | L | 156 | 3.9 |
| Medial orbitofrontal gyrus, anterior cingulate cortex rostral division | L | 98 | 4.5 |
| Inferior parietal gyrus, supramarginal gyrus, banks of the superior temporal sulcus | R | 200 | 4.4 |
| **Nulliparous > 12 weeks postpartum (p < .001)** |  |  |  |
| Superior temporal gyrus, banks of the superior temporal sulcus, middle temporal gyrus | L | 195 | 4.0 |
| Superior temporal gyrus, transverse temporal gyrus | L | 182 | 4.1 |
| Superior parietal gyrus | L | 152 | 4.0 |
| Medial orbitofrontal gyrus, anterior cingulate cortex rostral division | L | 135 | 4.4 |
| Inferior parietal gyrus, supramarginal gyrus, banks of the superior temporal sulcus | R | 182 | 4.3 |
| **Nulliparous > 24 weeks postpartum (p < .001)** |  |  |  |
| Superior parietal gyrus, inferior parietal gyrus | L | 107 | 4.1 |
| Superior temporal gyrus | L | 97 | 4.1 |
| Inferior parietal gyrus, supramarginal gyrus, banks of the superior temporal sulcus | R | 117 | 3.9 |

Note. P. = pars.

Table S16. Quality control measure (image quality rating (IQR)) of longitudinal measurements in mothers.

| subject | t0 | t1 | t2 | t3 | t4 | t5 |
| --- | --- | --- | --- | --- | --- | --- |
| sub01 | 80.59% | 81.61% | 81.62% | 81.78% | 81.41% | 81.44% |
| sub02 | 83.79% | 82.25% | 82.14% | 81.68% | 81.28% | 81.68% |
| sub03 | 80.12% | 79.72% | 79.76% | 78.77% | 79.03% | 78.99% |
| sub04 | 83.05% | 82.94% | 82.33% | 82.45% | 82.34% | 82.38% |
| sub05 | 80.98% | 81.58% | 81.68% | 81.41% | 80.43% | 80.70% |
| sub06 | 82.75% | 80.57% | 81.42% | 81.44% | 81.31% | 81.08% |
| sub07 | 80.78% | 80.50% | 79.97% | 80.97% | 79.94% | 79.59% |
| sub08 | 82.87% | 81.24% | 80.43% | 81.24% | 82.32% | 81.72% |
| sub09 | 80.17% | 79.60% | 81.03% | 80.58% | 80.81% | 80.26% |
| sub10 | 83.32% | 81.54% | 81.14% | 80.99% | 81.30% | 80.84% |
| sub11 | 82.81% | 83.08% | 82.44% | 81.89% | 80.71% | 83.59% |
| sub12 | 78.98% | 82.29% | 82.19% | 81.79% | 81.86% | 81.29% |
| sub13 | 80.06% | 81.29% | 81.64% | 81.46% | 81.44% | 81.07% |
| sub14 | 84.50% | 84.38% | 83.99% | 83.17% | 82.23% | 82.79% |
| sub15 | 84.12% | 82.48% | 83.09% | 83.07% | 82.61% | 82.72% |
| sub16 | 81.92% | 80.72% | 82.13% | 82.12% | 82.22% | 80.59% |
| sub17 | 83.64% | 81.81% | 81.80% | 81.77% | 81.00% | 81.85% |
| sub18 | 80.04% | 80.40% | 80.58% | 80.82% | 80.51% | 80.42% |
| sub19 | 79.53% | 80.61% | 81.03% | 80.78% | 81.24% | 80.60% |
| sub20 | 83.79% | 84.37% | 83.27% | 83.46% | 83.20% | 83.39% |
| sub21 | 81.20% | 82.00% | 82.27% | 81.95% | 81.67% | 81.94% |
| sub22 | 82.35% | 80.58% | 80.90% | 81.01% | 81.39% | 81.13% |
| sub23 | 82.72% | 81.85% | 81.61% | 81.94% | 81.72% | 81.90% |
| sub24 | 83.57% | 82.24% | 80.45% | 80.61% | 79.71% | 79.36% |

Note. IQR = image quality rating (combining noise vs. signal contrast and bias field correction quality), per time point: t0 = within 10 days of childbirth, t1 = 3 weeks postpartum, t2 = 6 weeks postpartum, t3 = 9 weeks postpartum, t4 = 12 weeks postpartum, t5 = 24 weeks postpartum.

*Figure S3.*

*Figure S4.*

**Supplementary Figure Legends**

Figure S1. **Correlation matrix of the associations between sex steroid and neurotransmitter receptor maps and maternal gray matter volume (GMV) across the first 24 weeks postpartum compared to nulliparous women.** The results depict the spearman rho per correlation with colors indicating the strength of the association, and statistically non-significant results left blank (grey) for visual clarity. NR3C1/NR3C2 = nuclear receptor subfamily 3, group C, member 1 and 2, respectively; ESR1 = Estrogen Receptor 1; PGR = progesterone receptor; OXTR = oxytocin receptor; GABA_A_ = GABA_A_ receptor; mGluR5 = metabotropic glutamate receptor 5.

Figure S2. **Association of maternal Pleasure in Interaction (PiI) with cerebellar GMV at three and six months postpartum (pp.).** **A** PiI at 12 weeks pp.is negatively associated with maternal brain volume in one cerebellar cluster **B** At 12 weeks pp. For the significant cluster, volume is represented by the mean value per participant, with a trendline (dotted). Correlation with the MPAS subscale PiI is depicted. **C** PiI at 24 weeks pp.is negatively associated with maternal brain volume in one cerebellar cluster. **D** At 24 weeks pp. For the significant cluster, volume is represented by the mean value per participant, with a trendline (dotted). Correlation with the MPAS subscale PiI is depicted. ↑ = positive association, ↓ = negative association; * the correlation coefficients (*r*) are significant at the *p* < .05 level.

Figure S3. **Development of cortical thickness in the postpartum (pp.) period. A** Increase in cortical thickness in postpartum women across the study period. **B** Greater cortical thickness in nulliparous than postpartum (pp.) women at childbirth and the last study timepoint of 24 weeks pp.

* using a *p* < .001 cluster-forming threshold and a *p* < .05 cluster-level FWE correction.

Figure S4. **Development of sulcus depth across the postpartum (pp.) period.** Increases in sulcus depth in postpartum (pp.) women between proximal time points from childbirth to three weeks, three weeks to six weeks and six weeks to nine weeks postpartum, and between the distal time points from nine to 24 weeks postpartum.

* using a p < .001 cluster-forming threshold and a p < .05 cluster-level FWE correction.
