# Supplementary figures and images for: Maternal brain alterations based on neurotransmitter and hormone receptor distributions over six months postpartum
Source: Transl Psychiatry. 2026 May 22;16:280. doi: 10.1038/s41398-026-04104-4 (PMC13197414; doi:10.1038/s41398-026-04104-4)

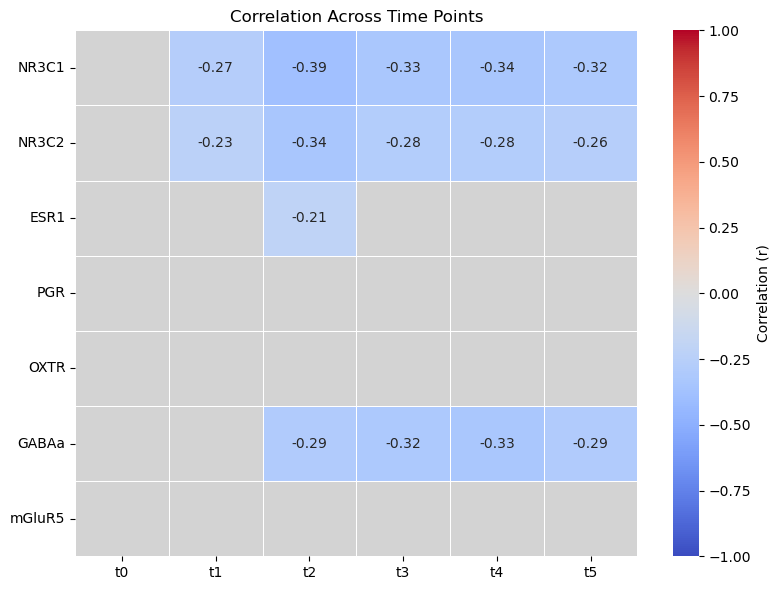

Supplement: Supplementary file 2 — Figure S1 [file 41398_2026_4104_MOESM2_ESM.png]

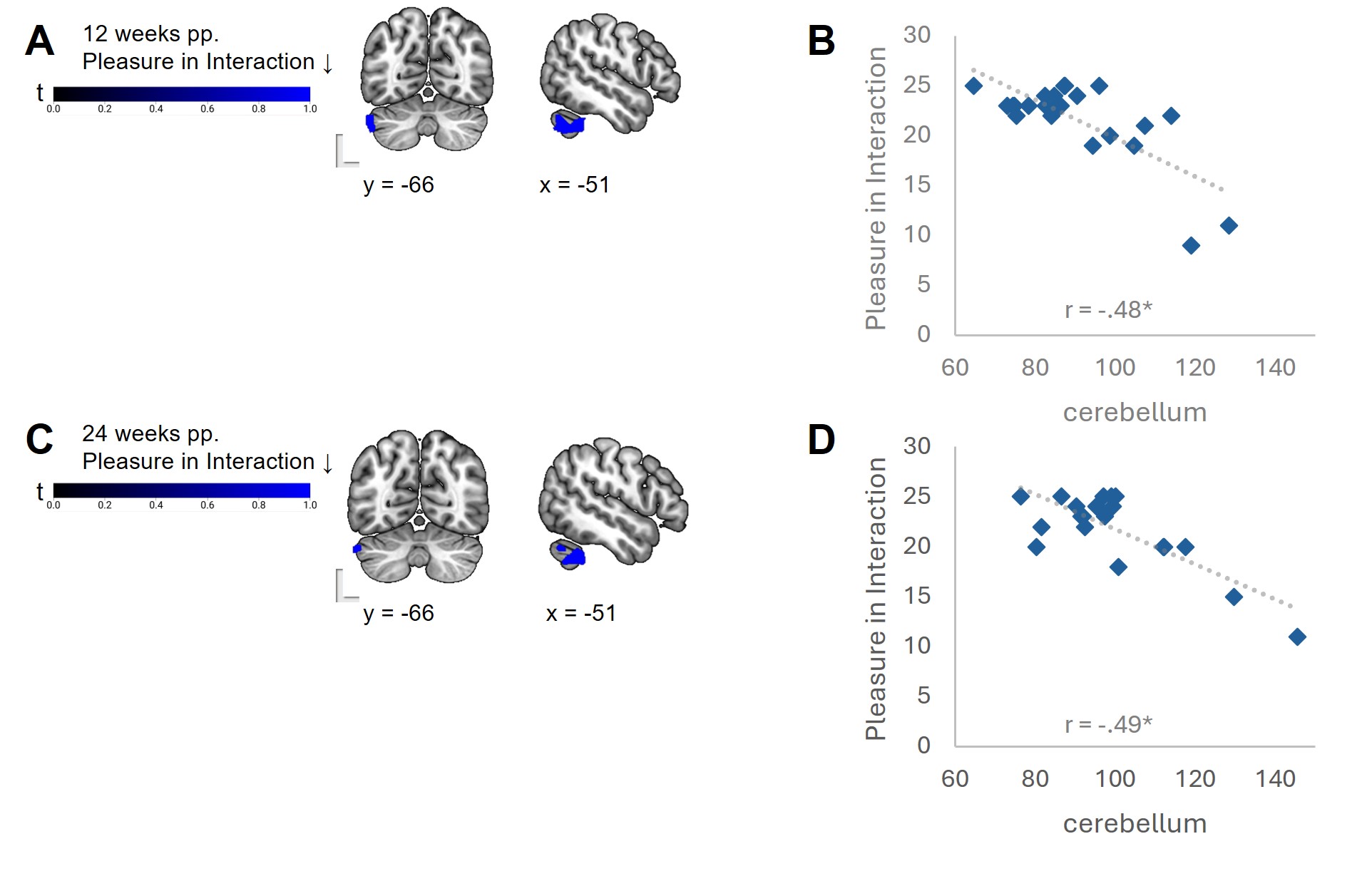

Supplement: Supplementary file 3 — Figure S2 [file 41398_2026_4104_MOESM3_ESM.jpg]

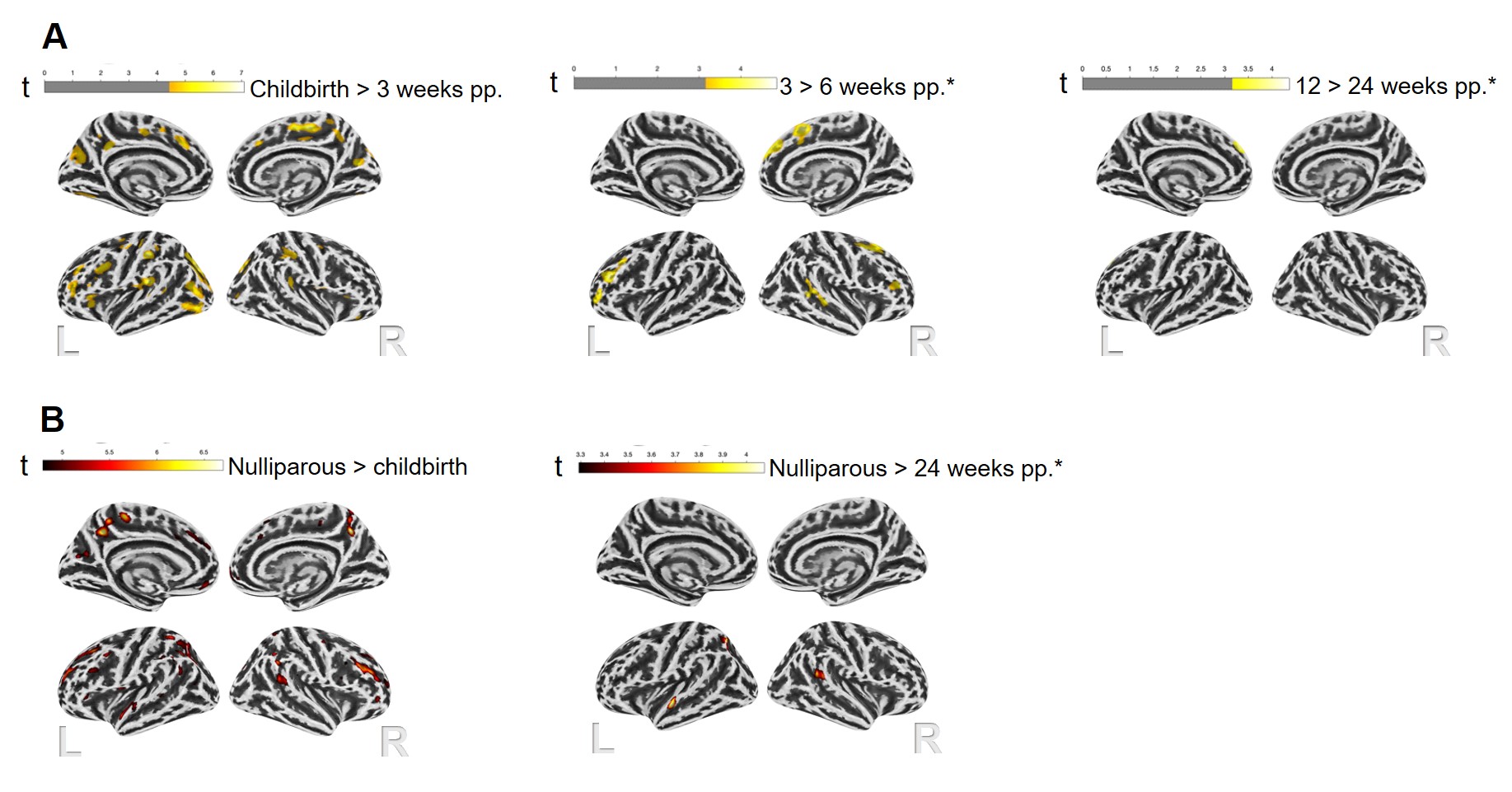

Supplement: Supplementary file 4 — Figure S3 [file 41398_2026_4104_MOESM4_ESM.jpg]

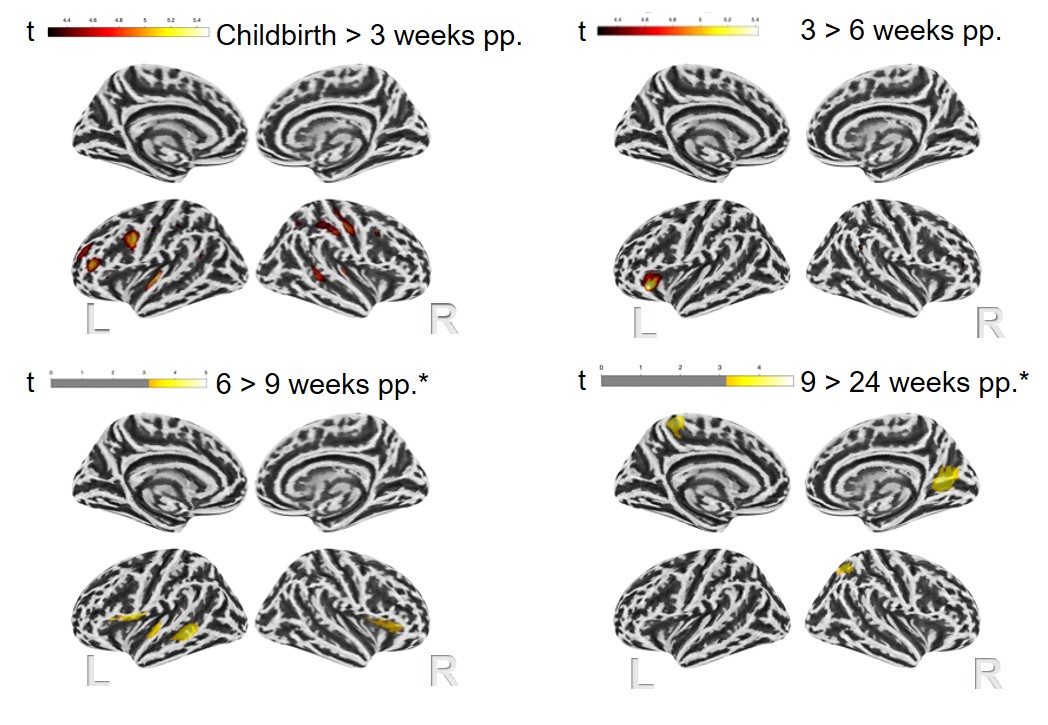

Supplement: Supplementary file 5 — Figure S4 [file 41398_2026_4104_MOESM5_ESM.jpg]
